# Functional Analysis of an Acid Adaptive DNA Adenine Methyltransferase from *Helicobacter pylori* 26695

**DOI:** 10.1371/journal.pone.0016810

**Published:** 2011-02-09

**Authors:** Arun Banerjee, Desirazu N. Rao

**Affiliations:** Department of Biochemistry, Indian Institute of Science, Bangalore, India; New England Biolabs, Inc., United States of America

## Abstract

HP0593 DNA-(N^6^-adenine)-methyltransferase (HP0593 MTase) is a member of a Type III restriction-modification system in *Helicobacter pylori* strain 26695. HP0593 MTase has been cloned, overexpressed and purified heterologously in *Escherichia coli.* The recognition sequence of the purified MTase was determined as 5′-GCAG-3′and the site of methylation was found to be adenine. The activity of HP0593 MTase was found to be optimal at pH 5.5. This is a unique property in context of natural adaptation of *H. pylori* in its acidic niche. Dot-blot assay using antibodies that react specifically with DNA containing m6A modification confirmed that HP0593 MTase is an adenine-specific MTase. HP0593 MTase occurred as both monomer and dimer in solution as determined by gel-filtration chromatography and chemical-crosslinking studies. The nonlinear dependence of methylation activity on enzyme concentration indicated that more than one molecule of enzyme was required for its activity. Analysis of initial velocity with AdoMet as a substrate showed that two molecules of AdoMet bind to HP0593 MTase, which is the first example in case of Type III MTases. Interestingly, metal ion cofactors such as Co^2+^, Mn^2+^, and also Mg^2+^ stimulated the HP0593 MTase activity. Preincubation and isotope partitioning analyses clearly indicated that HP0593 MTase-DNA complex is catalytically competent, and suggested that DNA binds to the MTase first followed by AdoMet. HP0593 MTase shows a distributive mechanism of methylation on DNA having more than one recognition site. Considering the occurrence of GCAG sequence in the potential promoter regions of physiologically important genes in *H. pylori*, our results provide impetus for exploring the role of this DNA MTase in the cellular processes of *H. pylori*.

## Introduction

Enzymatic DNA methylation is an important biochemical process that imprints DNA with new information. The sequence-specific post-replicative base methylation by DNA methyl-transferases (MTases) places the methyl group in the major groove of the DNA, without impeding Watson-Crick base pairing of adenine or cytosine. This mark helps in crucial DNA-protein interactions [1]. DNA methylation is catalyzed by S-adenosyl-L-methionine (AdoMet)-dependent DNA MTases. Prokaryotic DNA MTases are usually components of restriction-modification (R-M) systems that enable cells to resist propagation of foreign genomes that would otherwise kill them [2]. Based on the position of methyl group transfer on bases in DNA, DNA MTases are classified into two groups, exocyclic or amino MTases, and endocyclic or ring MTases [3]. The amino MTases methylate exocyclic amino nitrogen to form either N^6^-methyladenine or N^4^-methylcytosine, whereas the ring MTases methylate ring carbon to form C^5^-methylcytosine.

N^6^-methyladenine is mostly found in the genomes of bacteria, archaea, protists and fungi [4]. Methylation of the amino group of adenine lowers the thermodynamic stability of DNA [5] and alters DNA curvature [6]. Such structural effects can influence DNA–protein interactions, especially for proteins that recognize their cognate DNA-binding sites by both DNA primary sequence and DNA structure [7]. All exocyclic MTases have nine highly conserved motifs and are further subdivided into six groups, namely α, β, γ, ζ, δ, and ε, which are characterized by a distinct linear order of the AdoMet binding region (F*X*G*X*G), target recognition domain (TRD), and catalytic motif (DPPY) [3], [8]. TRDs are long stretches of amino acid sequence with poor conservation. The low amino acid sequence similarity between TRDs explains the specificity of a particular MTase for a specific sequence [9].

Analysis of the genome sequences of *H. pylori* strains 26695, J99, HPAGI, and G27 revealed an abundance of R-M systems [10]–[13]. Genome analysis of strain 26695 showed the presence of three putative Type III R-M systems and *hp0592-hp0593* constitute one such system. Based on the conserved motif arrangement, HP0593 MTase belongs to the β-subgroup of MTases. The amino acid sequence of HP0593 MTase has 38% identity and 55% similarity to EcoP1I MTase while it shows 33% identity and 50% similarity to EcoP15I MTase both of which belong to Type III R-M system [14].

Nobusato *et al.*, [15] showed that the gross genome polymorphism was linked with R-M gene homologues in *H. pylori*. One such example of gross polymorphism was shown to be linked with a Type III R-M system, in which a 5037 bp long DNA segment carrying *hp0592* and *hp0593* appears to have replaced a 2010 bp long DNA sequence. Ang *et al.*, [16] and Wen *et al.*, [17] carried out studies to delineate the changes in gene expression in response to exposure of *H. pylori* to different levels of external pH using genomic microarrays of *H. pylori*. This study demonstrated a two-fold over-expression of *hp0593* gene at pH 4.5. Chang *et al*., [18] showed that in *hp0017* (*virB4* homologue) knock-out mutant of *H. pylori*, *hp0593* showed more than 4-fold decreased expression. In general such studies implicate that this MTase could have essential functions in bacterial physiology. These observations therefore, give an impetus in exploring the possible roles played by this MTase in context to the physiology of *H. pylori.*

The present study reports biochemical characterization of HP0593, which is a functional Type III adenine MTase from *H. pylori.* Most interestingly, HP0593 MTase was found to be active at a pH optimum of 5.5. Overall this study focuses on the biochemical analysis of HP0593 MTase from *H. pylori* and provides insights into the mechanism of enzyme action.

## Materials and Methods

### Bacterial strains and plasmids

*E. coli* DH5α [F′ *end* A1 *hsd* R17 (r_k_^−^ m_k_^−^) *gln*V44 *thi*1 *rec*A1 *gyr*A (nal^R^) *rel*A1 Δ (*lacI*ZYA – *arg*F) U169 *deo*R (Φ80d*lac* Δ (*lacZ* M15)] cells were used for isolation of DNA. Proteins were expressed in BL21 (DE3) pLysS [F^−^ *omp*T hsdS_B_ (r_B_^−^ m_ B_^−^) *gal dcm* (DE3) pLysS (cam^ R^)] cells by transforming with appropriate plasmid constructs. *H. pylori* 26695 (*cagA^+^ iceA1 vacAs1am1*) strain used for isolating genomic DNA was a kind gift from NEB, USA.

### Enzymes and chemicals

Restriction and modifying enzymes were purchased from New England Biolabs, USA. *S-*Adenosyl-L-[^3^H] methionine, (67.3 Ci/mmol) was purchased from PerkinElmer Life Sciences, Singapore and GE healthcare Biosciences Ltd., Hong Kong. Chloramphenicol, bovine serum albumin, MgCl_2_, MnCl_2_, CoCl_2_, CaCl_2_, NiCl_2_, ZnCl_2_, ampicillin, imidazole, Coomassie Brilliant Blue R-250, RNase A, glutaraldehyde, and *S*-adenosyl-L-homocysteine (AdoHcy) were procured from Sigma-Aldrich Co., USA. Ni^2+^-NTA agarose beads were purchased from Invitrogen, USA. DE81 anion-exchange filter papers were purchased from Whatman, USA. All reagents used were of analytical or ultra-pure grade. Primers and duplex DNAs used in this study (Tables 1 and 2) were purchased from MWG, Germany and Sigma-Aldrich Co., USA. Double-stranded DNA concentration was measured spectrophotometrically, assuming an *A*_260_ of 1.0 corresponds to 50 µg/ml for double-stranded DNA and 33 µg/ml for single-stranded DNA. [γ*-^32^P*] ATP (4200 Ci/mmol) was purchased from Bhabha Atomic Research Centre, India.

**Table 1:** Primers used for cloning and mutagenesis.

Sr.No.	Primers used in this study (5′-3′)	RestrictionSite (+/-)
**1**	AT**CCATGG**ATATGCTTTTAAAGAATTTCCCGCAAACGATA	**Forward** **(+NdeI)**
**2**	AAT**GGATCC**ATCAATACACCACGCTTAAACGCTC	**Reverse** **(+BamHI)**
**3**	TACATTGATCG**CCCGGG**AACACTGGAAAC	**Y107G** **(+SmaI)**
**4**	GCGCATTCGTTATCGTCAATGCTGATA	
**5**	TACTCTGAA**CCCGGG**CAAAAACAACTC	**C54G** **(+SmaI)**
**6**	GCGCATTCGTTATCGTCAATGCTGATA	

The restriction enzyme site is indicated in bold faced letters and underlined.

**Table 2:** Duplex DNA used in this study.

Duplex	Duplex DNA sequence
1	5′ GGTCAGAGACCAGCAGTCCCTAAGT 3′ 3′ CCAGTCTCTGGTCGTCAGGGATTCA 3′
2	5′GATCAATAGATGC**GCAG**ATCATTTACATT 3′ 3′CTAGTTATCTACG**CGTC**TAGTAAATGTAA 5′
3	5′ TAGGTCAGAGACCA**GCAG**TCCCTAAGTAGCC 3′ 3′ ATCCATTCTCTGGT**CGTC**AGGGATTCATCGG 5′
4	5′ GCCGTGATCACCAAT**GCAG**ATTGACGAACCTTTGCCCACGT 3′ 3′ CGGCACTAGTGGTTA**CGTC**TAACTGCTTGGAAACGGGTGCA 5′
5	5′ GATCACTCCAT***A*** **CAG**GGTACAGTGGAG 3′ 3′ CTAGTGAGGTA***T*** **GTC**CCATGTCACCTC 5′
6	5′ CCTCGATCTC**GCT****GAGAGGATCCGACTAC** 3′ 3′ GGAGCTAGAG**CG***A*C**TCTCCTAGGCTGATG** 5′
7	5′ GATCAATAGAT**GCGC**AGATCATTTACATT 3′ 3′ CTAGTTATCTA**CGCG**TCTAGTAAATGTAA 5′
8	5′ GATCAATAGATCT**GCAGCT**CATTTACATTCG 3′ 3′ CTAGTTATCTAGA**CGTCGA**GTAAATGTAAGC 5′
9	5′ GATCAATAGAT**CTGCAG**ATCATTTACATTCG 3′ 3′ CTAGTTATCTA**GACGTC**TAGTAAATGTAAGC 5′
10	5′ GCTACAAGACCCTTC**GCAG**GGATCCGCGCGATCATG 3′ 3′ CGATGTTCTGGGAAG**CGTC**CCTAGGCGCGCTAGTAC 5′
11	**CH_3_**|5′ GCTACAAGACCCTTC**GCAG**GGATCCGCGCGATCATG 3′ 3′ CGATGTTCTGGGAAG**CGTC**CCTAGGCGCGCTAGTAC 5′
12	5′ GCTACAAGACCCTTC**GCAG**GGATCCGCGCGATCATG 3′ 3′ CGATGTTCTGGGAAG**CGTC**CCTAGGCGCGCTAGTAC 5′|**CH_3_**
13	**CH_3_**|5′ GCTACAAGACCCTTC**GCAG**GGATCCGCGCGATCATG 3′ 3′ CGATGTTCTGGGAAG**CGTC**CCTAGGCGCGCTAGTAC 5′|**CH_3_**
14	**CH_3_**|5′ GCTACAAGACCCTTC**GCAG**GGATCCGCGCGATCATG 3′ 3′ CGATGTTCTGGGAAG**CGTC**CCTAGGCGCGCTAGTAC 5′
15	**CH_3_**|5′ GCTACAAGACCCTTC**GCAG**GGATCCGCGCGATCATG 3′ 3′ CGATGTTCTGGGAAG**CGTC**CCTAGGCGCGCTAGTAC 5′|**CH_3_**
16	5′ **[Bt]CGATGCGACA**GCAG**ACCTCTAGTCCAGCGAAGACAGCAGACCTCTAG3′ 3′ GCTACGCTGT**CGTC**TGGAGATCAGGTCGCTTCTGT**CGTC**TGGAGATC 5′**
17	5′ CGATGCGACAGCAGACCTCTAGTCCAGCGAAGACA**GCAG**ACCTCTAG 3′ 3′ GCTACGCTGT**CGTC**TGGAGATCAGGTCGCTTCTGT**CGTC**TGGAGATC 5′
18	5′ **[Bt]**GATCAATAGATGC**GCAG**ATCATTTACATT 3′ 3′ CTAGTTATCTACG**CGTC**TAGTAAATGTAA 5′
19	5′ GATCAATAGATCCCCGGATCATTTACATT 3′ 3′ CTAGTTATCTAGGGGCCTAGTAAATGTAA 5′

The boldfaced region of the duplex represents the HP0593 MTase recognition sequence. Italicized and underlined bases represent HP0593 MTase recognition sequence perturbation. Restriction enzyme sites are shown as underlined. Methylated bases have been indicated by CH_3_. **[Bt]**, biotinylated duplex.

### General recombinant techniques

Restriction enzymes, DNA polymerases, T4 DNA ligase and T4 polynucleotide kinase were used according to manufacturers’ recommendations. Ligations, transformations and DNA electrophoresis were performed as described before [19]. Plasmid DNA pET14b and pUC19 were prepared as described earlier [19].

### Cloning, over-expression and purification of HP0593 MTase

The 1797 bp long *hp0593* gene was amplified from genomic DNA of *H. pylori* 26695 strain by polymerase chain reaction with Pfu polymerase using primers 1 and 2 (Table 1). The primers were designed with the help of the annotated complete genome sequence of *H. pylori* 26695 [10], identifying the putative gene sequence of *hp0593*, obtained from TIGR. The amplified PCR fragment was cloned into bacterial expression vector pET14b at NdeI and BamHI sites.

*E. coli* BL21 (DE3) pLysS cells were transformed with the pET14b-*hp0593* DNA using the standard protocol [19]. Individual colonies obtained after transformations were inoculated into 4.0 ml LB broth containing 70 µg/ml ampicillin and grown overnight. 1% of this primary inoculum was then used for reinoculation and grown to an A_600_ of 0.6. 2 ml of this uninduced culture was aliquoted out and HP0593-(His)_6_-tagged protein production was induced by the addition of 1.0 mM IPTG (isopropyl-1-thio-β-D-galactopyranoside). After 8 hrs of incubation at 18°C, the culture was cooled on ice and approximately equal numbers of bacterial cells present in uninduced and induced cultures were harvested by centrifugation at 5000 *g* for 10 min. The induction of the protein was checked by 0.1% SDS-10% polyacrylamide gel electrophoresis of crude cell extract obtained by sonication in 1X SDS-PAGE buffer containing dye. As controls, inductions were checked in only *E. coli* BL21 (DE3) pLysS cells and the same cells containing only pET14b vector.

*E. coli* BL21 (DE3) pLysS cells harboring pET14b-*hp0593* construct were grown in 600 ml of LB broth containing 70 µg/ml ampicillin to an A_600_ of approx 0.6. HP0593 protein expression was induced by the addition of IPTG to a final concentration of 1.0 mM, at 18°C. After 8 hrs of induction at 18°C, the culture was cooled on ice, and cells harvested by centrifugation at 6,000 *g* for 30 mins at 4°C. All the purification steps were carried out at 4°C. The purification of HP0593-(His)_6_-tagged protein was performed with Ni^2+^-NTA (nickel-nitrilotriacetic acid) affinity chromatography under native conditions. Briefly, the cell pellet was resuspended in sonication buffer (10 mM Tris-HCl pH 7.5, 0.2 mM EDTA, 100 µg/ml lysozyme, 150 mM NaCl, 10 mM β-mercaptoethanol, 5 mM imidazole and 10% glycerol), and lysed by sonication. The sonication process was carried out on ice and care was taken not to exceed the temperatures above 4°C. The cell lysate was centrifuged for one hour at 10,000 *g* at 4°C. The fusion protein was bound to a Ni^2+^-NTA resin equilibrated with buffer A (10 mM Tris-HCl pH 7.5, 0.2 mM EDTA, 150 mM NaCl, 10 mM β-mercaptoethanol, 5 mM imidazole and 10% glycerol). After thorough washing, proteins were eluted with a step-wise increase in imidazole (30 mM–300 mM). The purity of the protein was checked on 0.1% SDS-10% PAGE. Fractions containing homogeneous enzyme, were pooled and dialyzed against buffer B (10 mM Tris-HCl pH 7.5, 0.2 mM EDTA, 150 mM NaCl, 10 mM β-mercaptoethanol, and 10% glycerol) and aliquots of the purified protein were stored at −20°C. Protein concentration was estimated by Bradford’s method [20].

### Western blotting

Western blot analysis was carried out as described earlier [21]. Briefly, the protein sample was transferred onto nitrocellulose membrane by semi-dry transfer technique [19]. The blot was incubated with 1∶40,000 dilution of primary antibody (rabbit polyclonal anti-HP0593 antibody) and 1∶10,000 dilution of secondary antibody (goat anti-rabbit IgG-HRP conjugated). Finally, the blot was developed by addition of solution containing 10 mM Tris (pH 7.4), 0.6 mg/ml di-amino benzidine and 100 µl hydrogen peroxide.

### Two-Dimensional polyacrylamide gel electrophoresis (2-D PAGE)

Two-dimensional polyacrylamide gel electrophoresis (2-D PAGE) was performed according to the method of O'Farrell [22]. In the first dimension, 2.0 µM protein was resolved according to its isoelectric point (pI) by isoelectric focusing (IEF) using Biorad ReadyStrip™ 7 cm IPG strips, pH 3–10 range at 800 V for 17 h. In the second dimension, proteins are separated according to their approximate molecular weight using 0.1% SDS-10% polyacrylamide slab gel. The gel was electrophoresed by the use of a BioRad Protean II apparatus and was stained with Coomassie Brilliant Blue dye. The resolved protein band was subjected to in-gel digestion with trypsin followed by MALDI-TOF mass spectrometry for identification of peptide fragments.

### MALDI-MS analysis of HP0593 MTase

The purified protein was subjected to MALDI-MS analysis for molecular mass determination. MALDI-MS data were acquired on an Ultraflex TOF/TOF spectrometer (Bruker Daltonics, Billericia, MA, USA and Bremen, Germany), equipped with 50 Hz pulsed nitrogen laser (*l*¼337 nm), operated in positive ion reflectron mode using a 90-ns time delay, and a 25 kV accelerating voltage. The samples were prepared by mixing an equal amount of peptide (0.5 ml) with matrices dihydroxybenzoic acid/α-cyano-4-hydroxycinnamic acid saturated in 0.1% trifluoroacetic acid and acetonitrile (1∶1, v/v). Masses below 500 m/z were not considered due to interference from the matrix.

### Gel filtration analysis of HP0593 MTase

Native molecular mass of HP0593 MTase was determined by gel filtration chromatography analysis. A Superose™ 6 HR 10/30 column (bed volume∼24 ml) fitted into an AKTA basic 10 FPLC (GE Healthcare Life Sciences) was first calibrated with buffer B. The flow rate was maintained at 0.4 ml/min, and the elution profile was monitored by the absorbance at 280 nm. The void volume was determined using Blue Dextran, and the column was calibrated using following standard molecular-mass markers (Sigma): horse myoglobin (17 kDa), chicken ovalbumin (44 kDa), BSA (66 kDa), EcoP15I MTase (150 kDa), γ-globulin (158 kDa) and thyroglobulin (670 kDa). Different concentrations of HP0593 MTase were loaded (40–3200 µg/ml). The void volume (*V_o_*) of the column was found to be 7.5 ml. The elution volumes (*V_e_*) of marker proteins and HP0593 MTase were determined. The molecular mass of HP0593 MTase was calculated from the plot of *V_e_*/*V_o_ versus* log of molecular weight.

### Chemical cross-linking of HP0593 MTase

HP0593 MTase (2 µM) was incubated on ice for 10 min. Increasing amounts of glutaraldehyde were then added to the protein solution to a final concentration range of 0.01–0.08%, and the mixture was further incubated on ice for 10 min. The reaction products were separated by electrophoresis on a denaturing polyacrylamide gel (0.1% SDS-10% PAGE) and visualized by silver-staining. Furthermore after the cross-linking reaction, reaction products were subjected to MALDI-MS for molecular weight determination.

### Methylation assays

#### A. Filter binding assay

All methylation assays monitored incorporation of tritiated methyl groups in to DNA by using a modified ion-exchange filter binding assay [23]. Methylation assays were carried out in a reaction mixture containing duplex 2 DNA (Table 2) or pET14b or pET145-*hp0593* DNA (wherever mentioned), [*methyl*-^3^H] AdoMet, HP0593-(His)_6_-tagged protein in a standard methylation buffer (10 mM potassium phosphate pH 5.5, 7 mM β-mercaptoethanol, and with or without 1.0 mM MnCl_2_ wherever mentioned). After the incubation of enzyme and substrate at 37°C for 10 minutes, reactions were stopped by snap-freezing in liquid nitrogen and then aliquots were transferred onto small Whatman DE81 filter paper discs. Filter discs were washed, air dried, and the tritium content was determined using Beckman LS 6000 IC liquid scintillation counter. All data are corrected for nonspecific binding of [^3^H] AdoMet to the washed filter. Background counts were subtracted and data were analyzed. The counts were converted into nanomolar of methyl groups transferred to duplex DNA per min. All assays were done in triplicates and the average values were reported.

#### B. Biotin-avidin microplate assay

A biotin–avidin microplate assay was used for the quantitative analysis of enzymatic methylation of DNA [24]. MTase activity of HP0593 MTase was monitored by incorporation of [^3^H] methyl groups in biotin-tagged duplex 16 DNA (Table 2) containing the MTase recognition site. Typically, the reactions were performed with duplex DNA, purified HP0593 MTase, and [^3^H] AdoMet at 25°C for 10 min. Each experiment was done in duplicates, repeated three times, and the average values reported. Variations were in the range of 5–10%.

### Dot blot assay for methylation activity

Methylation activity was measured in a dot blot assay using rabbit primary antibodies raised against DNA modified with N^6^ methyl-adenine (NEB). To investigate methylation by HP0593 MTase, 250 picomol of duplex 2 DNA containing one 5’-GCAG-3’ site was incubated with 1.5 µM [^3^H] AdoMet and 1 µM purified protein in methylation buffer and incubated for 30 min at 37°C separately followed by protein inactivation at 95°C for 10 min. DNA was purified and spotted onto a poly-(vinylidene difluoride) (PVDF) membrane (Immobilon-N; Millipore, Billerica, MA, USA) and fixed by UV crosslinking (1.2 mJ/cm^2^ for 30 sec). Dot blot assay was performed as described earlier [25].

### Fluorescence spectroscopy analysis of HP0593 MTase-AdoMet interaction

Fluorescence spectroscopy analysis of HP0593 MTase-AdoMet interaction was carried out based on the intrinsic fluorescent signal of tryptophan. Fluorescence emission spectra and fluorescence intensities were measured for HP0593 MTase on a Shimadzu, RF 5000 spectrofluorimeter using a 1-cm stirred quartz cuvette at 37°C. The emission spectra were recorded over a wavelength of 300–400 nm with an excitation wavelength of 280 nm. HP0593 MTase was allowed to equilibrate for 2 min in methylation buffer before measurements were made. Aliquots of cofactor (final concentration 0.5 µM–6.25 µM) were added to HP0593 MTase (2 µM), and spectra were recorded. Each spectra recorded was an average of three scans. The fluorescence intensities were plotted against the total AdoMet concentration. The data were analyzed using the modified Stern–Volmer equations. The Stern–Volmer relationship is represented by *F*_0_/(*F*_0_ −*F*)  = 1/*{* [Q] · *f*_a_ · *K*_Q_ *}* +1/*f*_a_, where, *f*_a_ is the fractional number of fluorophores accessible to quencher, and *K*_Q_ is the quenching constant. The dissociation constants were calculated graphically using the modified Stern-Volmer plot (a plot of *F_0_*/(*F_0_* - *F*) *versus* 1/[Q]), where *K_Q_*  = 1/*K_d_* [*26*]. *F_0_* and *F* are fluorescence intensities in the absence and presence of cofactor respectively, Q is the quencher (AdoMet) concentration, *K_Q_* is the quenching constant and *K_d_* is the apparent dissociation constant.

### DNA binding studies by electrophoretic mobility shift assay

To assess the DNA binding ability of HP0593 MTase, electrophoretic mobility shift assays (EMSA) were performed as described [27]. Briefly, 29 mer duplex 2 DNA having 5’-GCAG-3’ site or duplex DNA without 5’-GCAG-3’ site (duplex 19), labeled with [γ-^32^P] ATP was incubated for 10 minutes on ice in binding buffer (10 mM potassium phosphate pH 5.5, 7 mM β-mercaptoethanol, and 10% (v/v) glycerol), in presence of HP0593 protein and/or AdoHcy or sinefungin. The reaction volumes were typically 10 µl. These were electrophoresed on 8% polyacrylamide gels in 0.5 X TBE buffer (89 mM Tris, 89 mM boric acid, 1 mM EDTA, pH 8.0). Electrophoresis was done at 4°C and at 90 V for seven hours. Gels were dried and protein-DNA complexes were visualized using Fuji PhosphorImager.

### Kinetic Studies

#### A. Determination of Kinetic Constants

Kinetic studies were done using a 29 mer duplex DNA (duplex 2) having a single 5’-GCAG-3’ as a recognition site for HP0593 MTase. Methylation assays were carried out as described earlier. In a series of similar reactions containing HP0593 MTase (150 nM) and [*methyl*-^3^H] AdoMet (1.5 µM), the concentration of DNA was varied in the range of 100–1000 nM. The velocities were fitted into a one-site binding (hyperbola) equation as follows:
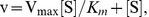
(equation 1)A non-linear regression analysis of initial velocity versus DNA concentration allowed the determination of *K*_m (DNA)_ and *V*_max_. The turnover number (*k*_cat_) was calculated as the ratio of *V*_max_ to the enzyme concentration used. Data were plotted by nonlinear regression analysis (curve fit) using Graphs Pad Prism software (version 5). Similarly, initial velocity experiments were carried out by varying the concentration of [^3^H] AdoMet in the range of 0.25 µM–4.0 µM while keeping a fixed DNA concentration of 5.0 µM and 150 nM HP0593 MTase at 37°C for 10 minutes. Hill equation was used to analyze the number of AdoMet molecules bound to the HP0593 MTase [28]. 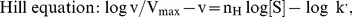
(equation 2)Where, k’ =  apparent dissociation constant, and n_H_  =  Hill Coefficient.

#### B. Preincubation studies

Preincubation studies were carried out by incubating 1.5 µM HP0593 MTase with either 1.5 µM duplex 2 DNA (Table 2) or 1.5 µM [^3^H-methyl] AdoMet at 25°C for 5 min. The reaction was initiated by adding AdoMet or DNA, respectively. At 10-, 20-, 30-, 40-, 50-, 60-, 75-, 90-, 120-s time intervals, 10-µl aliquots were removed and analyzed for product formation using the DE81 filter binding assay. In a control experiment, AdoMet and DNA were added together, and the reaction was started with HP0593 MTase. The experiment was done in duplicates and data was plotted using Graphs Pad Prism (version 5).

#### C. Isotope partitioning analysis

HP0593 MTase (1.0 µM) was preincubated with [^3^H] AdoMet (1.5 µM) at 25°C for 5 min. The preincubated reaction mixture was brought to a final volume of 200 µl with methylation buffer containing 1.5 µM [*methyl*-^3^H] AdoMet and 1.0 µM duplex 2 DNA. Aliquots of 20 µl each were removed at 10-, 20-, 30-, 40-, 50-, 60-, 90-, 120-, and 150-s time intervals, and the reaction was stopped by snap-chilling the samples in liquid nitrogen. Samples were then analyzed for radiolabeled product formation using a DE81 filter binding assay. In a parallel reaction, the above-mentioned preincubated mix was brought to 200 µl with methylation buffer containing 1.5 µM unlabeled AdoMet and 1.0 µM DNA, and the reaction was carried out as described earlier. The final concentrations of HP0593, DNA and AdoMet were 0.050 µM, 1.0 µM and 1.5 µM, respectively. The experiment was done in duplicates and data was plotted using Graphs Pad Prism (version 5).

#### D. Processivity studies

To determine the mode of methylation by HP0593 MTase, a 51 mer duplex 16 DNA (Table 2) with biotin-tag at 5’ end that contains two HP0593 MTase recognition sites (5’-GCAG-3’) separated by 21 bp was used. A master mix (400 µl) containing 2.0 µM biotin-conjugated 51 mer duplex (duplex 16, Table 2) and 150 nM of HP0593 MTase was preincubated at room temperature for 5 min to facilitate formation of the HP0593-DNA catalytic complex. After 5 min of preincubation, the reaction mixture was split in two. To one half 1.5 µM of [^3^H] AdoMet was added and aliquots of 20 µl were withdrawn at different time intervals (at 15-, 30-, 45-, 60-, 120-, 180-, 240-, and 300-s time intervals) and the reactions were stopped by snap chilling the samples in liquid nitrogen. To the remaining half of the reaction mixture, 1.5 µM of [^3^H] AdoMet and 20 µM of 51 mer untagged duplex DNA (duplex 17) containing two HP0593 sites was added as a trap. Aliquots of 20 µl were withdrawn at different time intervals up to 5 min. The samples were analyzed using biotin-avidin microplate assay as described earlier.

#### E. DNA binding studies-Surface plasmon resonance (SPR) spectroscopy analysis

The binding kinetics of purified HP0593 MTase with duplex 18 DNA was determined by surface plasmon resonance spectroscopy using the BIACORE 3000 optical biosensor (GE Healthcare Life-sciences, Uppsala, Sweden). A 5’-biotinylated 29 mer duplex (duplex 18, Table 2) with one HP0593 MTase recognition sequence was immobilized on a streptavidin-coated SA sensor chip (GE Healthcare Life-sciences, Uppsala, Sweden) as per the manufacturer’s recommendations. The binding reactions were carried out at 25°C in a continuous flow of buffer containing 10 mM HEPES, pH 7.4, 100 mM NaCl, 0.05% surfactant P-20 and with or without 1.0 mM MnCl_2_ at a flow rate of 20 µl/min. Increasing concentrations of HP0593 MTase (25–100 nM) in presence of 1.0 mM MnCl_2_ and HP0593 MTase (100–200 nM) in absence of metal were injected onto the surface of the biosensor chip for 120 sec at a flow rate of 20 µl/min followed by a dissociation period of 120 sec. The surface was regenerated by passing 5 µl of 0.05% SDS followed by 10 µl of the running buffer for further binding reactions. One of the four surfaces not having the biotinylated duplex DNA was used as a negative control. The background nonspecific binding and bulk concentration of HP0593 MTase was experimentally determined by simultaneous injection over a surface that lacked DNA. Each experiment was repeated thrice. The affinity and kinetic parameters (rate constants) were determined by subjecting the sensorgrams of association and dissociation phases to global analysis using BIAevaluation software version 3.0. The global fitting analyzes both association and dissociation data for all concentrations simultaneously using 1∶1 Langmuir binding model.

### Site-directed mutagenesis

Site-directed mutagenesis was performed using PCR based technique to replace required amino acids [29]. Mutations were introduced into the *hp0593* gene by using the two stage megaprimer PCR method. PCR reactions were carried out with Pfu DNA polymerase (Fermentas Life Sciences). For each substitution, a mutagenic primer and appropriate second primer was used. In the first round of PCR, appropriate oligonucleotide primers (Table 1) and pET14b-*hp0593* DNA were used to amplify a DNA fragment, which was used as a megaprimer in the second round of PCR. The full-length PCR product was obtained in the second round PCR by extension of the megaprimer. The PCR product, thus obtained was purified, digested with DpnI restriction enzyme to cleave the methylated template DNA, transformed into *E. coli* DH5α strain and plated on LB agar media containing ampicillin (50 µg/ml). The mutagenic primers were designed in such a way to change the respective amino acids and to create a Type II restriction enzyme site. Hence, the resultant plasmids could be screened easily. The resultant plasmids were used for expression and purification of mutant HP0593 proteins. Amino acid (shown in bold) in the catalytic motif DPP**Y** was replaced by using primers 3 and 4 (primer 3 was mutagenic). By substituting **Y** with **G**, it was possible to introduce convenient restriction enzyme site (SmaI), thus allowing screening of mutants. The mutant was confirmed by restriction digestions and by DNA sequencing. Similarly, amino acid (shown in bold) in the catalytic motif P**C**Q was replaced by using primers 5 and 6 (primer 5 was mutagenic), where **C** (shown in bold) was substituted with **G** and a SmaI restriction enzyme site was created for screening of mutants. The mutants were confirmed by DNA sequencing. The resultant plasmids were used for expression and purification of mutant HP0593 proteins was carried out as described earlier.

### Miscellaneous methods

For checking the purity, protein samples were separated on 0.1% SDS-10% polyacrylamide gels according to the method described by Laemmli [30]. Polyclonal antiserum was generated against (His)_6_-tagged HP0593 MTase in rabbits following standard protocol [19].

### Statistical analysis

Each experiment was performed in duplicates or triplicates and repeated at least twice. Data are shown as mean ± SE. All the analyses were done with Graphs Pad Prism (version 5) or Sigma plot (version 9).

## Results and Discussion

### Multiple sequence alignment between HP0593 MTase, EcoP1I MTase and EcoP15I MTase

**Figure 1:**
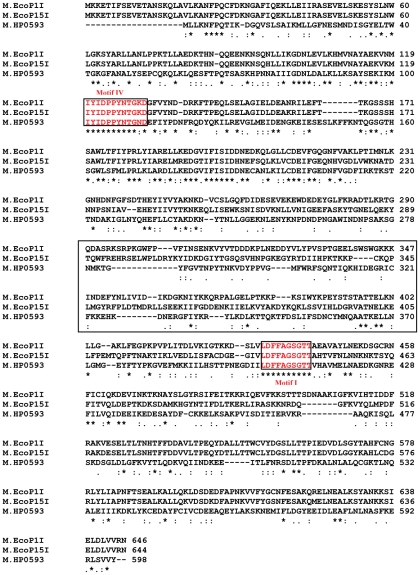
Multiple sequence alignment between HP0593, EcoP1I and EcoP15I MTases. (*) represents sequence identity and (:) represents sequence similarity. The differences exist in the target recognition domain (TRD), which is shown by a box. The amino acid sequences of conserved motifs (motif IV and motif I) are boxed.

Fig. 1. shows multiple sequence alignment between HP0593, EcoP1I and EcoP15I MTases carried out through ClustalW programme. It shows the sequence identity (*) and sequence similarity (:) between these sequences, which are a part of Type III R-M systems. From this analysis it is clearly evident that the differences exist in the target recognition domain (TRD) of these Type III MTases. The differences in TRD are responsible for different sequence specificity among these MTases.

### Cloning, over-expression, purification, and identification of HP0593 protein

A 1797 bp PCR product (Fig. S1A) representing *hp0593* gene was amplified from *H. pylori* genomic DNA using primers 1 and 2 (Table 1). The amplified product was cloned using NdeI and BamHI restriction enzyme sites into the expression vector pET14b. The authenticity of the clone was confirmed by restriction digestion pattern (Fig. S1B). A polypeptide of expected molecular weight (M_r_) was expressed at high levels upon induction using 1.0 mM IPTG (Fig. S1C). HP0593 was expressed as an N-terminal (His)_6_-tagged protein, and was purified to near homogeneity as judged from 0.1% SDS-10% polyacrylamide gel electrophoresis using silver staining (Fig. S1D). When purified HP0593 protein was checked on SDS-PAGE an anomalous migration was observed. While the theoretical M_r_ of the protein with the (His)_6_ is expected to be 70.4 kDa, the experimental M_r_ determined from SDS-PAGE was 66.0 kDa. Therefore, matrix-assisted laser desorption ionization mass spectrometry (MALDI-MS) was carried out with purified HP0593 and the profile showed a single peak with expected molecular mass of 70.6 kDa (Fig. S1E). The protein was identified as an adenine-specific DNA methyltransferase from *H. pylori* by peptide mass fingerprint analysis. The sequence coverage was ∼30% (data not shown). Western blot was carried out using polyclonal antibodies raised in rabbits against the purified HP0593 protein. The antibody specifically reacted with HP0593 and a single band was detected (Fig. S1F).

### Two-dimensional polyacrylamide gel electrophoresis (2-D PAGE)

**Figure 2:**
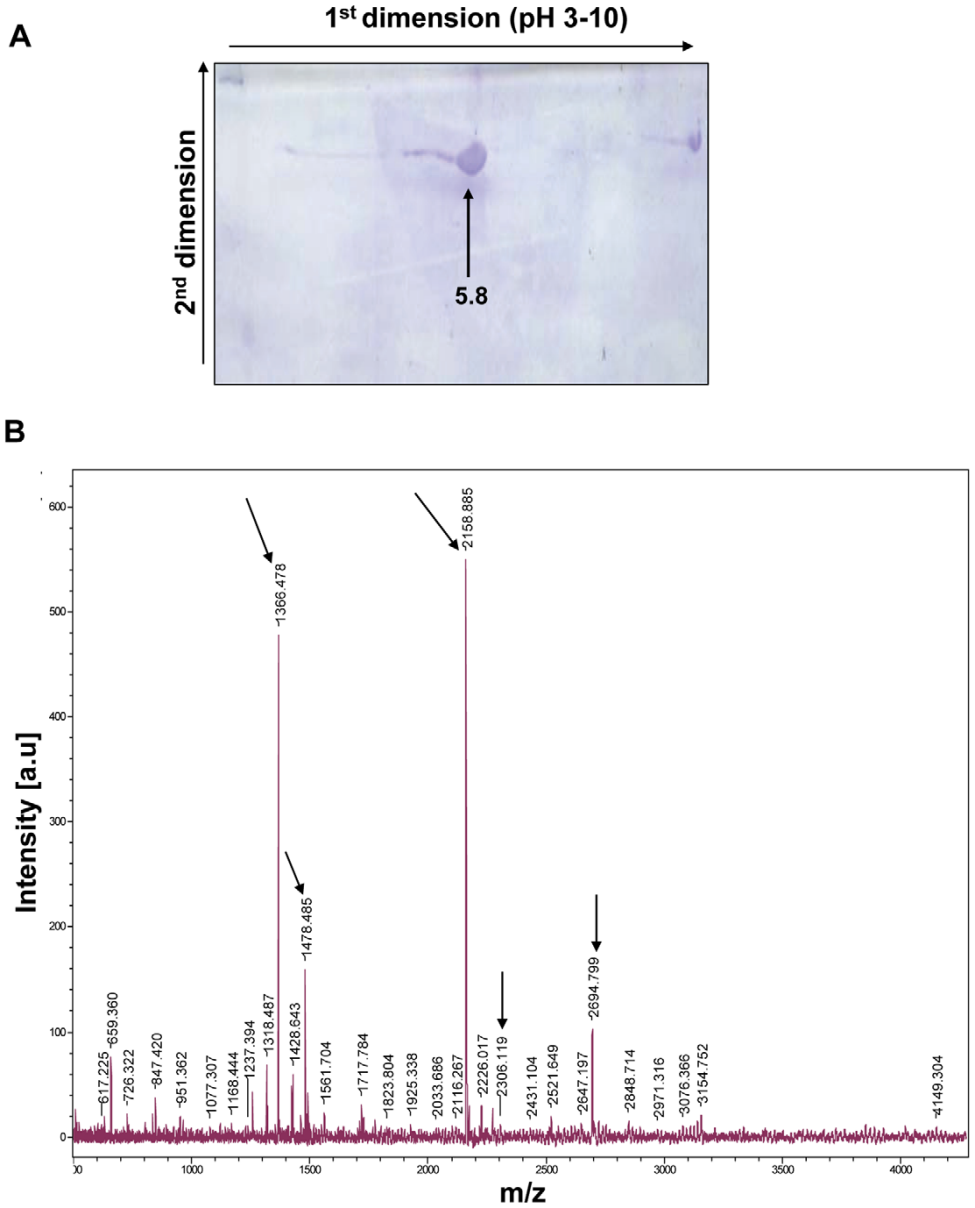
Characterization of (His)_6_-HP0593 recombinant protein. **A**. Two-Dimensional PAGE analysis of (His)_6_-HP0593 recombinant protein. The arrow indicates HP0593 protein, which migrates at a pI of ∼5.8. **B**. MALDI-TOF-MS analysis of tryptic digested pH 5.8 protein band. The matching peptide ion fragments are shown by arrows marked.

Most of the predicted proteins (∼70%) in *H. pylori* have a calculated pI >7.0 but the theoretical pI of HP0593 as predicted from protein sequence was found to be 5.12. Two-dimensional polyacrylamide gel electrophoresis (2-D PAGE) was carried out with Biorad ReadyStrip™ 7 cm IPG strips, with a pH range of 3–10, and the experimental pI of HP0593 protein was determined to be ∼5.8 (Fig. 2A). Furthermore, the protein band at pH 5.8 was excised from the gel, digested with trypsin and subjected to MALDI-TOF-MS analysis. Five peptide ions matched with the expected ions (Fig. 2B), confirming the authenticity of HP0593 protein.

### Oligomeric status and molecular mass determination of HP0593 MTase

Gel-filtration chromatography was performed to determine size and subunit structure of HP0593 MTase in solution. HP0593 MTase eluted as two asymmetric peaks at positions corresponding to a globular protein of ∼72 kDa and ∼144 kDa (Fig. S2A), suggesting that the enzyme exists both as monomer and dimer under native conditions. To confirm this observation, when increasing concentrations of purified HP0593 protein (40 µg/ml–3200 µg/ml) were subjected to gel-filtration both monomer (72 kDa) and dimer (144 kDa) peaks were obtained, although the monomer fraction was more compared to dimer fraction. The peak fractions were checked on 0.1% SDS-10% PAGE (data not shown), which showed the presence of HP0593 protein in both monomer and dimer fractions (inset Fig. S2A). Reinjection of the dimer peak resolved the protein into monomer and dimer populations, suggesting that these two forms exist in equilibrium (data not shown).

Glutaraldehyde is a homobifunctional cross-linking reagent that cross-links N-terminal primary amines of lysine residues, resulting in formation of amidine cross-links between protein subunits. Chemical cross-linking of HP0593 MTase with glutaraldehyde was carried out to determine the oligomeric nature of the enzyme. Glutaraldehyde-treated HP0593 MTase migrated with a relative molecular mass of 140 kDa (Fig. S2B). It was observed that increasing the concentration of glutaraldehyde in the cross-linking reaction resulted in an increase in the cross-linked HP0593 MTase. These results clearly demonstrate that HP0593 MTase exists both as monomer and dimer in solution. Furthermore, MALDI-MS analysis of 0.06% and 0.08% glutaraldehyde-treated HP0593 MTase confirmed the presence of both the monomer and dimer species of HP0593 MTase, respectively (data not shown).

Most of the characterized DNA MTases such as HhaI MTase [31], EcoRI MTase [23], and EcoDam MTase [32] exist as monomers in solution. However, MTases such as CcrM MTase [33], adenine MTases from DpnII R-M systems [34], EcoP15I MTase [35] and HaeIV MTase [36] exist as dimers in solution. RsrI MTase [37] and MspI MTase have been shown to dimerize at high protein concentrations [38]. In addition, β-class methyltransferases such as LlaCI MTase [39] and KpnI MTase [40] have been shown to dimerize in solution. In case of KpnI methyltransferase dimerization has been shown to be required for high affinity substrate binding needed for catalysis [41].

### Determination of sequence specificity of HP0593 MTase

**Figure 3:**
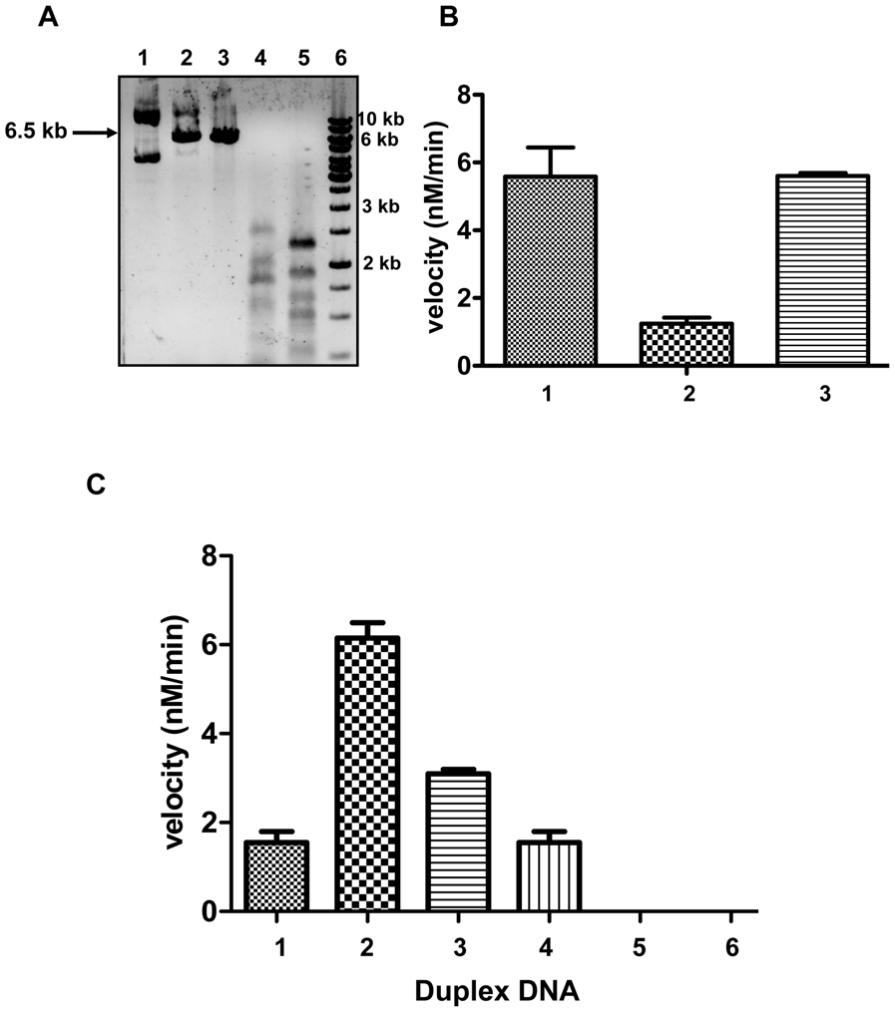
Sequence specificity of HP0593 MTase. **A.** Restriction digestion pattern of pET14b-*hp0593* plasmid DNA. Lane 1, DNA alone, lane 2, DNA + R.PstI; lane 3, DNA + R.PvuII; lane 4, DNA + R.HpyCH4V; lane 5, DNA + R.AluI; lane 6, 1.0 kb DNA ladder. **B.** Vector DNA methylation. Methylation reaction was carried out in presence of 2.5 µM of site (5’-GCAG-3’) concentration of pET14b DNA or pET14b-*hp0593* plasmid DNA or duplex 2 DNA, 150 nM HP0593 MTase and 1.5 µM [methyl-^3^H] AdoMet in methylation buffer at 37°C for 10 min. 1  =  pET14b DNA, 2  =  pET14b-*hp0593* plasmid DNA and 3  =  duplex 2 DNA (positive control). **C.** Methylation activity of HP0593 MTase in presence of duplex DNAs of varying length (25 bp to 41 bp). Duplexes 1–4 contain 5’-GCAG-3’ sequence, duplexes 5 and 6 contain 5’-ACAG-3’ and 5’-GCTG-3’ sequences, respectively. This experiment was done in duplicates.

Initial studies by Vitkute *et al.*, [14], predicted the sequence specificity of HP0593 MTase to be 5’-CTGCAG-3’ (isospecific to PstI MTase), due to resistance to cleavage by PstI restriction enzyme. However, the authors of this study suggested that the possibility of resistance to PstI restriction enzyme could be due to a hypothetical MTase of a lower specificity (5’ CTGCA 3’, 5’ TGCAG 3’, etc.). To confirm these observations pET14b-*hp0593* DNA was isolated from an expression strain after induction with IPTG. The DNA was checked for being sensitive or resistant to PstI (5’ CTGCAG 3’), HpyCH4V (5’ TGCA 3’), PvuII (5’ CAGCTG 3’) and AluI (5’ AGCT 3’) restriction enzymes. The digestion pattern clearly showed that pET14b-*hp0593* DNA was cleaved by PstI (1 site), PvuII (1 site), HpyCH4V (20 site) and AluI (18 sites) restriction enzymes (Fig. 3A). Hence, one could rule out that the sequence specificity of HP0593 MTase is neither isospecific to PstI, PvuII, HpyCH4 nor AluI. As can be seen from Fig. 3B methylation of pET14b-*hp0593* DNA was 3-fold less compared to pET14b DNA alone, which suggested a basal level of methylation in pET14b-*hp0593* DNA. Amino acid sequence comparision between HP0593 MTase and PstI MTase showed only 20% sequence identity and there was no identity or similarity in target recognition domains of the two MTases suggesting a difference in sequence specificity of HP0593 MTase and PstI MTase. To check the possibility that HP0593 MTase could recognize a tetranucleotide sequence (5’-GCAG-3’), a series of duplex DNAs were synthesized (duplexes 1 to 4) containing 5’-GCAG-3’ as a common sequence. Methylation activity was detected in all the above duplex DNAs (Fig. 3C). However, the level of methylation activity was different in case of the four duplexes tested here (duplexes 1–4). This could possibly be due to the different lengths of the duplexes and flanking sequences of the recognition site, which might affect the methylation activity. As a control when duplexes 5 and 6 were used, in which the 5’-GCAG-3’ sequence was changed to either 5’-**A**CAG-3’ or 5’-GC**T**G-3’, respectively, methylation activity was found to be negligible. These results clearly point out that 5’-GCAG-3’ is the recognition sequence for HP0593 MTase.

### Determination of site of methylation by HP0593 MTase

Amino acid sequence analysis revealed that other than the DPPY catalytic motif, HP0593 MTase contains a PCQ motif, which is the catalytic motif for C^5^-cytosine MTases. In order to determine, whether a C or A was the target base of methylation in 5’-GCAG-3’/3’-**C**GTC-5’ sequence, duplex 7 was used in which bottom strand 3’-cytosine (shown in bold and underlined) was first analyzed for methylation. Duplex 7 (5’-GCGCAG-3’/3’-CGCGTC-5’) harbors an overlapping site for HhaI MTase (5’-GCGC-3’) and HP0593 MTase (5’-GCAG-3’). HhaI MTase methylates the first C in 5’-G**C**GC-3’ sequence (shown in bold and underlined). When the duplex DNA was premethylated with HP0593 MTase, HhaI restriction enzyme (R.HhaI) cleaved the DNA (Fig. S3A), demonstrating that HP0593 MTase did not methylate the bottom strand 3’-cytosine. As a control reaction, when the same duplex DNA was premethylated with HhaI MTase, it showed protection against R.HhaI.

Similarly, to find out whether HP0593 MTase methylated the bottom strand 5’-cytosine (shown in bold and underlined) of 5’-GCAG-3’/3’-CGT**C**-5’ sequence, duplex 8 (5’-GCAGCT-3’/3’-CGTCGA-5’) was used in which AluI restriction enzyme site (5’-AGCT-3’/3’-TCGA-5’) overlapped with HP0593 MTase (5’-GCAG-3’) recognition site. Premethylation of the duplex DNA with HP0593 MTase did not show protection against AluI restriction enzyme, ruling out that the bottom strand 5’-cytosine was the site of methylation by HP0593 MTase (Fig. S3B).

Likewise, to check if the upper strand 5’-adenine (shown in bold and underlined) of 5’-GC**A**G-3’/3’-CGTC-5’ sequence was the target of methylation, duplex 9 was designed with overlapping PstI restriction enzyme (5’-CTGCAG-3’/5’-CTGCAG-3’) and HP0593 MTase recognition site (5’-GCAG-3’/3’-CGTC-5’). Premethylation of duplex DNA with HP0593 MTase and subsequent digestion with PstI restriction enzyme showed protection against PstI restriction enzyme (Fig. S3C). PstI restriction enzyme showed resistance against cleavage at 5’-CTGC**A**G-3’, if the adenine is methylated at N^6^ position. Based on this observation it was concluded that HP0593 MTase methylates the adenine in 5’-GCAG-3’ sequence. As a control reaction, when duplex 9 was digested with PstI restriction enzyme without any prior methylation by HP0593 MTase, it was cleaved.

In another experiment, a set of methylated duplex DNAs (duplexes 11–15) were synthesized, where 5’-GCAG-3’/3’-CGTC-5’ sequence was placed in the center in all duplex DNAs. Methylation activity was compared between methylated duplex DNAs (duplexes 11 to 15) and unmethylated duplex DNA (duplex 10). It is clear from Fig. S3D, that when upper strand cytosine was methylated (5’-G**C**AG-3’/3’-CGTC-5’) (duplex 11), or in duplex 12, where the bottom strand 3’-cytosine (5’-GCAG-3’/3’-**C**GTC-5’) was methylated, methylation activity was seen. When both upper and bottom strand cytosines (5’-G**C**AG-3’/3’-**C**GTC-5’) were methylated as in the case of duplex 13, the methylation activity was comparable to unmethylated duplex DNA. Taken together our results suggest that the upper strand or bottom strand cytosines (5’-G**C**AG-3’/3’-**C**GTC-5’) were not the sites of methylation by HP0593 MTase. When duplexes 14 and 15 were checked for methylation activity where adenines were methylated, no methylation activity could be detected. Taken together these data convincingly demonstrate that in the 5’-GCAG-3’sequence adenine is the target base of methylation by HP0593 MTase.

### Determination of N^6^ –adenine methyltransferase activity

**Figure 4:**
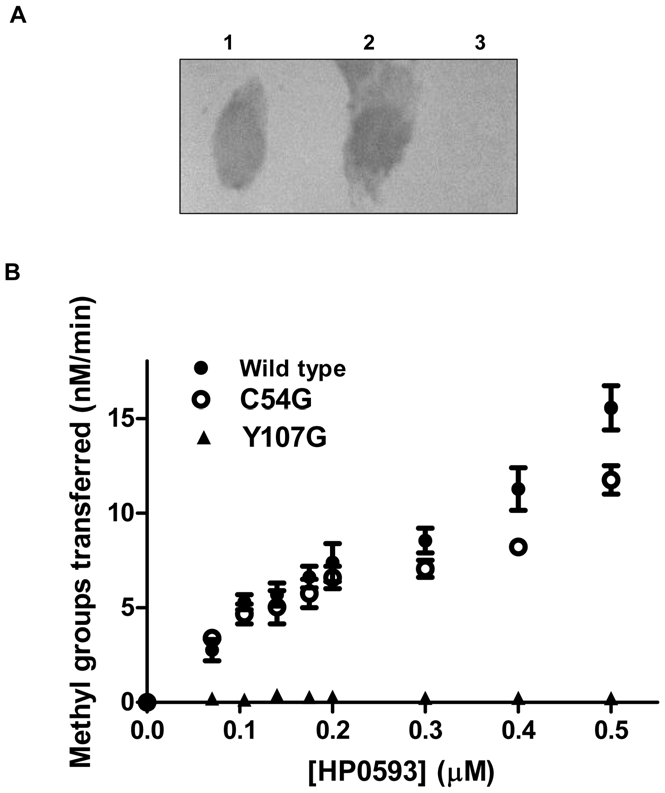
Determination of methylation activity. **A**. Dot blot assay. Lanes 1 and 2, HP0593 MTase methylated duplex 2 (duplicates); lane 3, unmethylated duplex 2. **B.** Characterization of HP0593 MTase C54G and Y107G mutants. Increasing concentrations of wild-type or Y107G or C54G mutants of HP0593 MTase (70–500 nM) were incubated with 5.0 µM duplex 2 and 1.5 µM AdoMet in methylation buffer at 37°C for 10 min. The reactions were stopped and analyzed as described in the materials and methods. (•) wild type, (○) C54G, (▴) Y107G.

To examine the enzymatic activity of HP0593 MTase, duplex 2 DNA (which contains a single 5’-GCAG-3’ site) was used for methylation with HP0593 MTase. The methylation status of the DNA was determined using N^6^ –methyladenine (m6A) specific antibody in a dot blot assay as described in the materials and methods. A positive signal was detected as m6A antibody interacted with methylated duplex 2 containing HP0593 MTase recognition site (Fig. 4A). No signal was detected when unmethylated duplex 2 was used, which served as a negative control.

### Purification and characterization of catalytic motif (Y107G and C54G) HP0593 mutant proteins

All N^6^-adenine methyltransferases have a conserved catalytic motif (D/N/S PP Y/F). Several groups have performed mutational studies on amino acids in these motifs, which in turn have revealed the significance of these motifs in catalysis [1], [3]. For instance, Pues *et al*. [42] have shown in the case of TaqI MTase that replacement of Y108 with alanine or glycine resulted in mutant MTases with reduced enzymatic activities, which shows the importance of tyrosine in the methylation activity. Mutational analysis of motif IV (DPPY) in EcoP1I MTase and EcoP15I MTase demonstrated the importance of tyrosine in catalytic activity [43]. Most interestingly, the amino acid sequence analysis of HP0593 MTase revealed the presence of PCQ like motif, which is the catalytic motif for C^5^–cytosine methyltransferases. In order to check the role of both these motifs, site-directed mutagenesis was performed to replace Y107 and C54 of HP0593 MTase by glycine. HP0593 Y107G and HP0593 C54G mutant proteins were purified to near homogeneity and analyzed on SDS-polyacrylamide gel wherein there was no change in mobilities (data not shown). Methylation activity of both the mutant proteins was analyzed as a function of increasing enzyme concentration. It was found that the Y107G mutant protein (Fig. 4B) was catalytically inactive as compared to wild-type HP0593 MTase. On the other hand the C54G mutant protein was found to be as active as the wild-type HP0593 MTase (Fig. 4B), indicating that HP0593 MTase is indeed an adenine MTase and not a C^5^- cytosine MTase.

### Substrate binding

**Figure 5:**
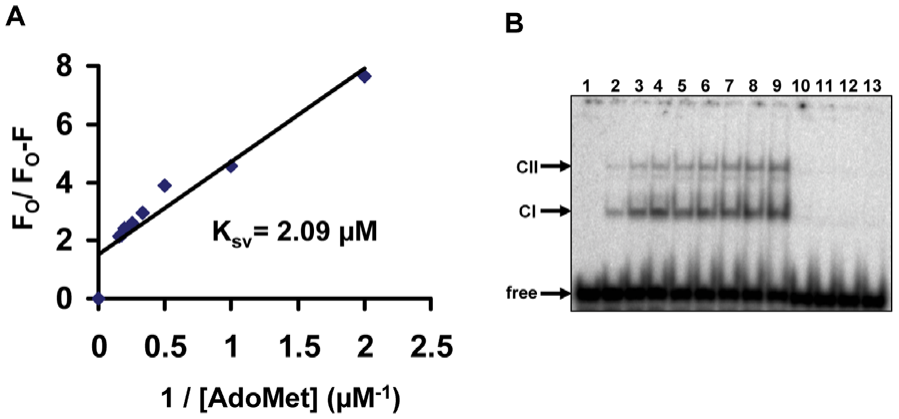
Binding of substrates to HP0593 MTase. **A**. AdoMet binding to HP0593 MTase: Modified Stern-Volmer plot for determining the *K*_SV_ AdoMet. **B.** Electrophoretic mobility shift assay: Binding of HP0593 MTase to duplex 2 DNA in presence of AdoHcy. Lane 1; radiolabeled duplex 2, lanes 2–9, increasing concentrations of HP0593 MTase (0.5 µM–5.0 µM) were incubated with 5’ [γ*-^32^P*] end-labeled duplex 2 (approximately 100 nM) in methylation buffer on ice for 10 min in presence of 5 µM AdoHcy and analyzed as described in materials and methods; lanes 10–13, chased with excess (5, 10, 15, and 20-fold, respectively) of unlabeled DNA (duplex 2).

HP0593 MTase has four tryptophans at positions 40, 163, 268 and 304, which provide a basis for probing ligand-induced conformational changes. The intrinsic fluorescence properties of HP0593 MTase were exploited in a fluorescence-quenching assay to determine the dissociation constant for AdoMet. Small aliquots of cofactor (final concentrations 0.5 µM–6.25 µM) were added to HP0593 MTase (2 µM), and spectra were recorded. The binding of AdoMet to HP0593 MTase resulted in quenching of fluorescence (data not shown). Each spectra recorded was an average of three scans. The Stern-Volmer plot for AdoMet showed a negative deviation from linearity, a result expected only when a fraction of the tryptophans are accessible to quenching by ligand binding (data not shown). Therefore, a modified Stern-Volmer plot was used to analyze the quenching [26]. The data in Fig. 5A shows a linear plot, suggesting that the cofactor binding is the dominant fluorescence-quenching phenomenon over the range of concentrations used (0.5–6.25 µM). The *K_SV_* (Stern-Volmer constant) value calculated from the modified Stern-Volmer relationship (equation 2) was found to be 2.09 µM. It is evident from Fig. 5A the intercept value is ∼ 1.85, which probably indicates more than one class of fluorophores (tryptophan in this case) involved in AdoMet binding.

DNA binding studies were carried out by electrophoretic mobility shift assay and performed as described [*2*7] using [γ-^32^P]-labeled 29mer duplex DNA (duplex 2) containing a single 5’-GCAG-3’ site. Binding reactions were carried out in 10 mM potassium phosphate buffer pH 5.5 containing 7 mM β-mercaptoethanol in the absence of divalent metal ions. 5 µM of AdoHcy, an end product of methylation reaction and 10 µM of sinefungin, a competitive inhibitor of all DNA MTases, were included separately with increasing amounts of HP0593 MTase in the binding buffer. Protein-DNA complexes were visualized by phosphoimager. Shifted complexes were seen in case of duplex 2 DNA and HP0593 MTase (Fig. S5A) and also in presence of AdoHcy (Fig. 5B) and sinefungin (Fig. S5B). In all three cases two different DNA-protein complexes were observed - a major fast-running complex 1 (CI) and a minor slow-running complex 2 (CII). As gel filtration chromatography experiments showed the presence of both monomer and dimer species of HP0593 protein, we surmise that the fast-running complex could be HP0593 monomer-DNA and the slow-running complex could be a HP0593 dimer-DNA complex. In EMSA done with DNA with specific recognition sequences (5’-GCAG-3’) (in the absence of cofactors) two bands of fast and slow migrating complexes were observed (Fig. S5A). These complexes were probably weak and therefore, not stable during electrophoresis. However, in the presence of cofactor (5 µM AdoHcy), band intensity of the two complexes were found to be greater compared to the EMSA done in the presence of DNA and HP0593 protein only. This suggested the formation of a stable ternary complex in the presence of AdoHcy. With non-specific duplex 19 DNA (lacking 5’-GCAG-3’ sequence), no complexes were formed even in the presence of cofactors (Fig. S5C). Based on the above observations we conclude that a specific interaction of HP0593 MTase occurs in presence of cognate DNA, which is stabilized by AdoHcy and in some extent by sinefungin.

### Kinetics of methylation

#### A. Determination of pH optimum for HP0593 MTase

**Figure 6:**
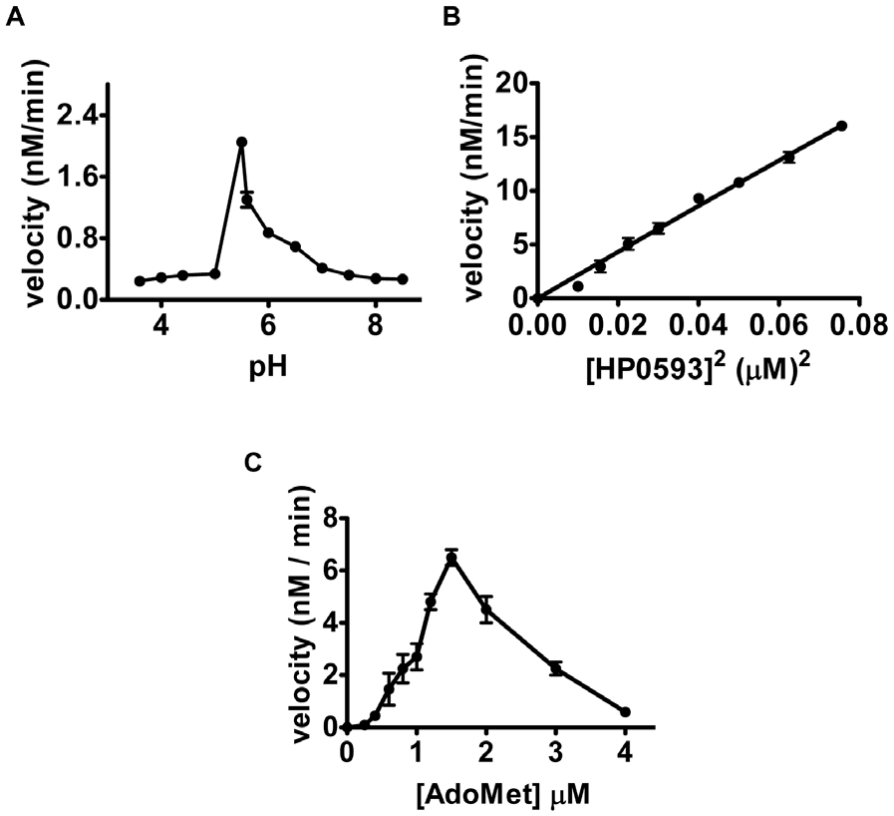
Kinetics of methylation. **A**. Determination of pH optima. Methylation activity of purified HP0593 MTase (50 nM) was measured in buffers with a pH range of 3.5 to 8.5 containing 2.5 µM of duplex 2, 1.5 µM [*methyl*-^3^H] AdoMet at 37°C for 10 min. Methylation activities were quantified by a filter-binding assay as described in materials and methods. **B**. Initial velocity versus square of HP0593 MTase concentration. Methylation assays were carried out in a reaction mixture containing 5 µM of duplex 2 DNA, 1.5 µM [methyl-^3^H] AdoMet and increasing concentrations of HP0593 MTase (20–300 nM) in methylation buffer at 37°C for 10 min. The samples were analyzed as described in materials and methods. Initial velocity data were plotted against the square of HP0593 MTase concentrations. **C**. Initial velocity vs. AdoMet concentration. Methylation assays were carried out in reactions containing 5 µM of duplex 2 DNA and increasing concentrations of [^3^H] AdoMet (0.25–4.0 µM) in methylation buffer at 37°C for 10 min. HP0593 MTase (150 nM) was added to start the reaction. Incorporation of methyl groups were estimated as described in materials and methods.

Most of the known DNA methyltransferases are catalytically active in the pH range of 7.0-8.5, but HP0593 MTase showed negligible activity in this pH range. Microarray experiments by Wen *et al.*, [17] had shown that *hp0593* gene shows a 2.5-fold over-expression at pH 4.5. As the natural niche of *H. pylori* is around pH 4.0–5.0, it is possible that HP0593 MTase could be active under acidic conditions. Methylation activity was therefore, monitored as a function of pH. The methylation reaction was carried out in presence of 50 nM HP0593 MTase, 2.5 µM of duplex 2, 1.5 µM [*methyl*-^3^H] AdoMet, and in buffers with a pH range of 3.5 to 8.5. When initial rates were plotted at different pH, the optimum pH for HP0593 methylation activity was found to be pH 5.5 with duplex 2 as the substrate (Fig. 6A). These observations suggested that HP0593 MTase possesses a unique property of being an acid-adaptive MTase. This may have an implication in acidic conditions, wherein HP0593 MTase might methylate sequences in the promoter regions, which in turn affect gene expression. Thus, HP0593 may play a crucial role in the physiology of *H. pylori.* It is noteworthy to mention that virus T1 DNA MTase shows optimum activity at pH 6.9 [44] while the archaebacterial PabI MTase is active at a pH range of 6.0 to 6.7 [45].

#### B. Rate of methylation versus enzyme concentration

To establish the relationship between the initial velocity of the reaction and enzyme concentration, the rate of DNA methylation catalyzed at different HP0593 MTase concentrations was determined. The substrate used for this analysis was 5.0 µM of duplex 2 having a single 5’-GCAG-3’ site. Varying concentrations (20 nM–300 nM) of HP0593 MTase were added to the reaction mixture containing duplex 2 DNA and 1.5 µM [*methyl*-^3^H] AdoMet and incubated at 37°C for 10 min. When initial velocities were plotted as a function of enzyme concentrations, a non-linear plot was obtained (data not shown). This indicated that at low concentrations of HP0593 MTase, the initial velocity of the reaction was not directly proportional to the enzyme concentration. Further, replotting of the initial velocity of the reaction against the square of HP0593 MTase concentrations yielded a linear plot (Fig. 6B). These results indicate that the cooperative binding of two molecules of HP0593 MTase is required to methylate DNA. This behavior has been observed for RsrI MTase [37] and KpnI MTase [40], which are dimers in solution.

#### C. Effect of DNA and AdoMet on the rate of reaction

Kinetic studies were done using duplex 2 having a single 5’-GCAG-3’ site. In a series of similar reactions containing HP0593 MTase (150 nM) and [*methyl*-^3^H] AdoMet (1.5 µM), the concentration of DNA was varied in the range of 100–1000 nM. When initial velocities were plotted against varying concentrations of duplex DNA, a rectangular hyperbola was obtained (data not shown), confirming that HP0593 MTase obeys Michaelis-Menten kinetics.

From non-linear regression analysis of initial velocity versus DNA concentration, *K*_m__(DNA)_ of 0.15±0.01 µM and V_max_ of 0.0017±0.06 µM/min was established. k_cat_ was calculated as 1.88×10^−4^ s^−1^. These values are in general agreement with the values of other DNA MTases. The *K*_m__(DNA)_ for EcoRV MTase with 20 mer duplex DNA was 0.3 µM and for KpnI MTase with pUC19 DNA was 0.15 µM. The k_cat_ for FokI MTase with 21 mer duplex was found to be 4.5×10^−4^ s^−1^, and for KpnI MTase with pUC19 DNA were 3.16×10^−4^ s^−1^ [3].

To study the effect of AdoMet on initial velocity of HP0593 MTase catalyzed reaction, methylation reactions were carried out with 150 nM HP0593 MTase, 5.0 µM duplex 2, and varying concentrations of AdoMet (0.25–4.0 µM) at 37°C for 10 min. When initial velocities were plotted against varying concentrations of AdoMet, a sigmoidal behavior was observed, and it was found that methylation reaction was inhibited above 1.5 µM of AdoMet (Fig. 6C). AdoMet saturation curve clearly showed more than one molecule of AdoMet cooperatively binds to HP0593 MTase. Hill plot [log [AdoMet] versus log (v/v_max_-v)] analysis of the above data revealed a Hill coefficient of 2.3 (data not shown), indicating that two molecules of AdoMet bound to the HP0593 MTase with respect to monomeric MTase concentration. More importantly, the substrate inhibition at higher concentrations of AdoMet clearly indicated that there could be more than one AdoMet binding site on the protein. However, with the present data it is difficult to distinguish whether both subunits of the enzyme bind to AdoMet or 2 molecules of AdoMet bind to one subunit cooperatively. Two molecules of AdoMet binding has been demonstrated in the case of PvuII MTase [46], T4 Dam MTase [47] and EcoDam MTase [48]. In case of EcoDam MTase, two AdoMet molecules were shown to have distinct roles- one as methyl group donor and other as an allosteric effector [48].

### Preincubation studies with HP0593 MTase

**Figure 7:**
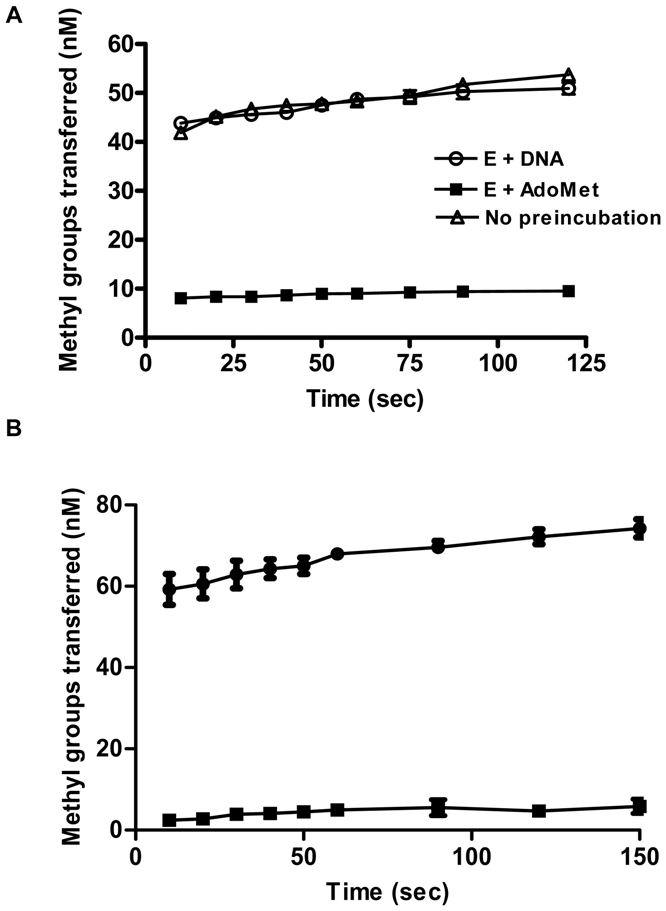
Preincubation and isotope partitioning analysis. **A**. Time course of methylation reaction under different preincubation conditions. HP0593 MTase was preincubated with either DNA (○) or [methyl-^3^H] AdoMet (▪) or without preincubation (Δ) as described in materials and methods. The reaction was then started with the addition of [methyl-^3^H] AdoMet or DNA, respectively. Aliquots were removed at the defined time points, and the incorporation of methyl groups measured by the filter binding assay as described in materials and methods. **B.** Isotope partitioning analysis of HP0593 MTase. Methylation assays were carried out in reaction mixture containing 1.0 µM HP0593 MTase, 1.0 µM DNA and 1.5 µM AdoMet. (-•-), Product formation after enzyme was preincubated at 25°C with high specific activity [methyl-^3^H] AdoMet (67.3 Ci/mmol) and reaction started with DNA and labeled [methyl-^3^H] AdoMet. (-▪-), Product formation after enzyme was preincubated with high specific activity [methyl-^3^H] AdoMet and reaction started with DNA and unlabeled AdoMet. The final concentrations of HP0593, DNA and AdoMet were 0.050 µM, 1.0 µM and 1.5 µM, respectively.

For the catalytic cycle of DNA MTases, binding of substrates could occur in a random or sequential order. To determine this, HP0593 MTase was preincubated with [*methyl*-^3^H] AdoMet or with duplex 2 for 5 min, and the reaction was initiated by addition of DNA or [*methyl*-^3^H] AdoMet, respectively. Under stoichiometric conditions, the order of preincubation of HP0593 MTase with either AdoMet or DNA had significant influence on the rate of product formation. The data in Fig. 7A, clearly demonstrates that the preformed enzyme-DNA complex was highly efficient than the preformed enzyme-AdoMet complex. One explanation for this would be that the preformed enzyme-AdoMet complex is not catalytically competent and that enzyme must dissociate, bind DNA, and then rebind AdoMet, which could slow down productive complex formation. In a control experiment, where reaction was carried out without any preincubation the product formation was found almost same as the reaction where enzyme-DNA was preincubated and reaction was started with adding AdoMet.

### Isotope partitioning analysis of HP0593 MTase

In order to confirm that the enzyme-AdoMet binary complex is not catalytically competent, an isotope partitioning experiment was performed. In an isotope partitioning experiment, a presaturated binary complex of HP0593 MTase with [methyl-^3^H] AdoMet was diluted into a mixture containing excess DNA and unlabeled AdoMet. This determines what fraction of the bound-labeled AdoMet would react before exchanging with unlabeled AdoMet. The formation of radiolabeled product reflects the propensity of the enzyme bound labeled substrate to undergo catalysis without dissociation [49]. Preincubation of HP0593 MTase with [^3^H] AdoMet (1.5 µM) resulted in a burst of product formation upon the addition of duplex 2 DNA and labeled AdoMet (Fig. 7B). The burst was followed by a constant rate of product formation. A decreased burst was observed when unlabeled AdoMet was used in the chase (Fig. 7B), which is evident by 6-fold decrease in vertical axis intercept. Burst magnitude is defined as the vertical axis intercepts resulting from extrapolation of the linear portion of the progress curve to zero time. These results indicated that the HP0593 MTase-AdoMet complex formed was not catalytically competent. This is because a chase including 1 µM duplex 2 DNA and 1.5 µM unlabeled AdoMet resulted in 6-fold less product formation. If AdoMet was required to bind to HP0593 MTase before DNA for catalysis, all of the prebound AdoMet would form catalytically productive complex. Isotope partitioning experiments have been used successfully to determine the catalytic competency of enzyme substrate complex and to decipher the order of binding as in the case of EcoRI MTase [50], HhaI MTase [51], MspI MTase [52], and KpnI MTase [40].

### Mode of methylation by HP0593 MTase on DNA having two recognition sites

In a processive mode of enzymatic catalysis, a single substrate binding event is coupled to multiple rounds of catalytic turnover. Non-processive catalysis occurs when enzyme dissociates from an initially bound substrate after the initial catalytic turnover. If HP0593 MTase methylates DNA having two sites in a non-processive manner, it would dissociate from the DNA after one site methylation, and would reassociate in the next round of catalysis to methylate the second site. If, on the other hand, the MTase methylates in a processive manner, it is expected that two methyl groups will be incorporated into a DNA molecule containing two sites, resulting in methylation of both sites in single binding event.

To determine if HP0593 MTase can methylate in a processive or distributive manner, a 51 mer biotin-tagged duplex 16 (having two recognition sites) was incubated in the presence of a limiting amount of enzyme and an excess of tritiated AdoMet. HP0593 MTase was preincubated with the duplex DNA for 5 min to promote formation of the HP0593-DNA catalytic complex. Following this incubation, the reaction mixture was split into two halves and to one half a 10-fold excess of untagged duplex DNA (duplex 17) was added. Reaction aliquots withdrawn at different time intervals were checked for methylation by capturing the biotinylated-methylated duplex DNA via avidin coated microplate and the unbound fraction by spotting directly on DE81 membrane. If HP0593 MTase works in a processive manner, then dilution with a competitor DNA should not inhibit further methylation. We found that in the presence of a 10-fold excess of nonbiotinylated DNA the extent of the methylation reaction did not increase (Fig. S4), but in the absence of nonbiotinylated competitor rapid additional methylation was observed (Fig. S4). Furthermore, to rule out any inhibition of HP0593 MTase catalyzed methylation reaction in presence of excess of nonbiotinylated DNA, a filter binding assay was carried with reaction mixture containing both biotinylated and non-biotinylated duplex DNA. The rate of methylation was almost similar in both the cases suggesting that there was no substrate inhibition in presence of excess of competitor duplex DNA (Fig. S4). Based on the above results, a distributive mechanism of methylation is indicative in which each cycle of methylation by HP0593 MTase resulted in incorporation of one methyl group.

The distributive mechanism of DNA methylation is a crucial adaptation of DNA MTases in R-M systems for the biological function of these systems. It is important that an endonuclease reaches its site on the phage DNA before it is modified. It therefore, makes sense that MTases are distributive, which considerably lower down the rate of DNA methylation, whereas endonucleases, in general, are processive in their mode of action. The distributive mechanism of DNA methylation of EcoRV MTase is a direct consequence of its order of substrate binding because AdoHcy cannot leave the ternary enzyme–DNA–AdoHcy complex and AdoMet cannot bind an enzyme–DNA complex [53].

### Effect of divalent metal ions on methylation activity of HP0593 MTase

**Figure 8:**
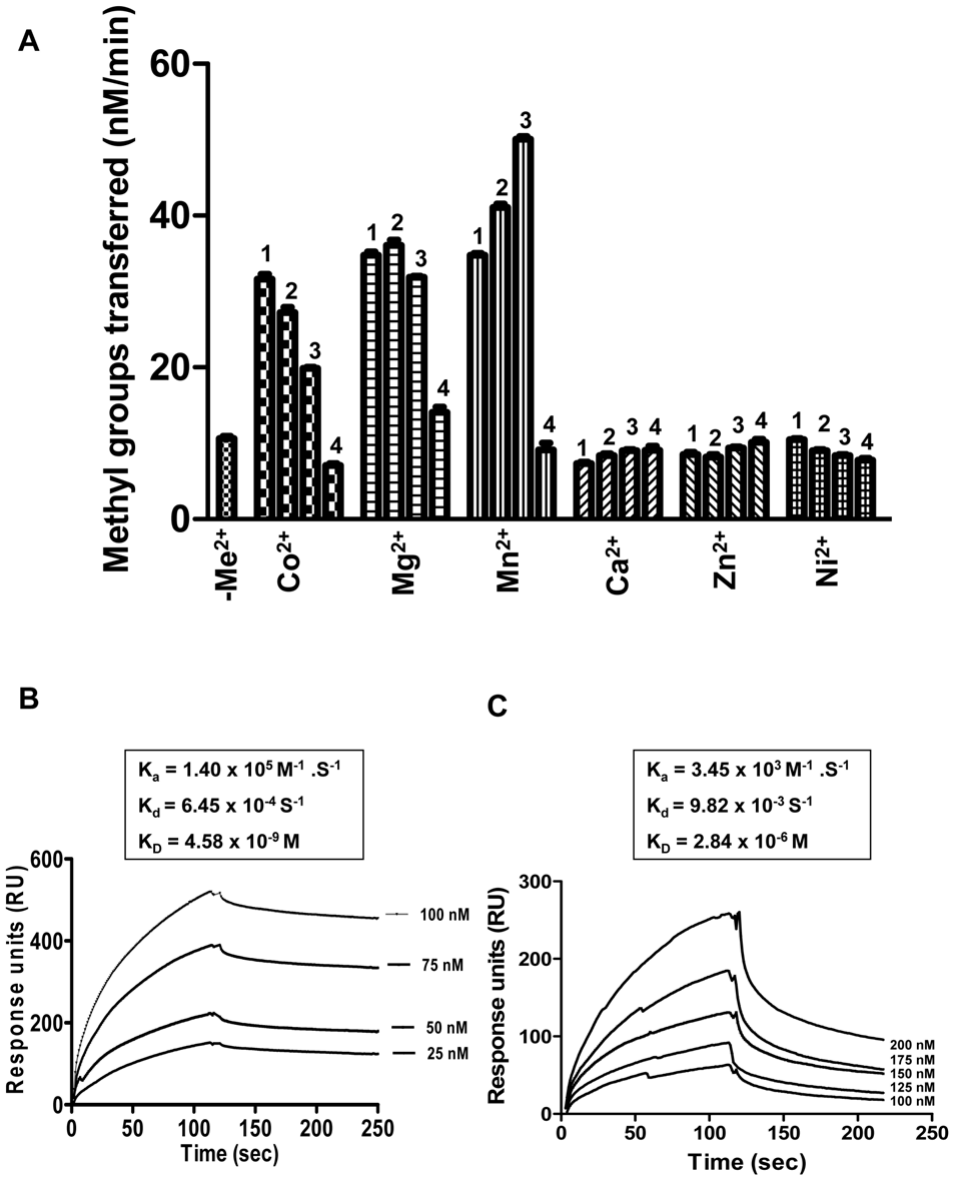
Effect of divalent metal ions on methylation activity of HP0593 MTase. **A**. Histogram showing methylation activity of 250 nM HP0593 MTase in the absence of any metal ions (-Me^2+^) and in the presence of cobalt chloride (Co^2+^), magnesium chloride (Mg^2+^), manganese chloride (Mn^2+^), calcium chloride (Ca^2+^), zinc chloride (Zn^2+^), and nickel chloride (Ni^2+^). 1 = 0.4 mM, 2 = 0.5 mM, 3 = 1.0 mM, and 4 = 5.0 mM. Kinetics of DNA binding. HP0593 MTase was injected for 120 sec over streptavidin chip containing immobilized duplex 18 DNA at a flow rate of 20 µl/min followed by dissociation phase of 120 sec. The global fit of the data was used to calculate the binding constants. **B.** SPR sensorgram displaying the response of increasing HP0593 MTase concentrations (25–100 nM) in presence of 1.0 mM MnCl_2_. (*Inset*) kinetic constants. **C.** SPR sensorgram displaying the response of increasing HP0593 MTase concentrations (100–200 nM) in absence of metal. (*Inset*) kinetic constants.

During the investigation of optimal reaction conditions for HP0593 MTase activity, it was found that divalent metal ions Mn^2+^, Mg^2+^, and Co^2+^ stimulated the methylation activity (5.0-fold, 3.5-fold and 3.0-fold, respectively) (Fig. 8A), whereas Ca^2+^, Zn^2+^, and Ni^2+^ ions do not affect the methylation activity of HP0593 MTase (Fig. 8A). MTase activities that are part of Type III R–M systems are known to require Mg^2+^ for their activity. Additionally, MTases within members of IIB, IIG and IIH R–M subtypes are stimulated by Mg^2+^ [54]. EcoP15I MTase, which is part of a Type III R–M system, requires Mg^2+^ for both base-flipping and methyl group transfer [55]. In the case of M.EcoP15I, magnesium ion is needed to stabilize the extra-helical base conformation. With regard to HP0593 MTase, metal ions are not essential for enzyme activity but stimulate the reaction. Addition of Ca^2+^ or Mg^2+^, but not Mn^2+^, Co^2+^ or Cd^2+^ to the reaction buffer stimulated the activity of the PspGI MTase [56]. Recently, Chan et al., [57] demonstrated that in case of HpyAV restriction endonuclease from *H. pylori*, activity was stimulated by Mn^2+^ and Co^2+^. Hence, it may be possible that Mn^2+^ and Co^2+^ may play an important role in the functioning of R-M system in context of physiology of *H. pylori*.

### Kinetics of DNA binding

Surface plasmon resonance spectroscopy was used to determine the kinetics of DNA binding for HP0593 MTase in absence and presence of metal. Surface plasmon resonance measures the change in refractive angle arising from a binding event. To monitor DNA-protein interaction, biotinylated DNA was immobilized on the surface of streptavidin (SA) chip and HP0593 protein was passed over the surface in increasing concentrations to allow determination of binding constants. The association and dissociation of the protein to DNA was monitored by changes in refractive index due to the binding event on the sensor surface. As Mn^2+^ showed maximum stimulation in methylation activity compared to other metal ions, the interaction between HP0593 MTase and 29 mer duplex 18 DNA that was biotinylated at 5’ end was investigated in absence or presence of 1.0 mM MnCl_2_ on SA sensor chip. The background nonspecific binding and bulk concentration of HP0593 MTase were experimentally determined and deducted by simultaneous injection over a surface that lacked DNA. Fig. 8B shows the sensorgram for interaction of HP0593 MTase with 29 mer duplex 18 DNA having one recognition site in presence of 1.0 mM MnCl_2_. It shows a significant increase in the response corresponding to the increasing concentrations of HP0593 MTase. The data was analyzed by global fit according to simple 1∶1 Langmuir equation by BIAevaluation software version 3, yielding a binding constant (K_D_) of 4.58×10^−9^ M, whereas HP0593 MTase-duplex DNA interaction in absence of metal ion yielded a binding constant of 2.84×10^−6^ M (Fig. 8C), which also correlated with EMSA results carried out in absence of metal. Hence, in presence of Mn^2+^ there was 1000-fold increase in affinity of HP0593 MTase with duplex DNA. So, manganese might increase the affinity of specific binding at the recognition sequence for HP0593 MTase, which was also evident by 5-fold stimulation in methylation activity. The K_D_ value determined here was in general agreement with the values of other MTase-DNA binary complexes such as the K_D_ value for KpnI MTase-DNA complex was 65 nM [40].

The key finding of the present study is the identification of an N^6^-adenine methyltransferase from *H. pylori*, which methylates the adenine in 5’-GCAG-3’ sequence at an optimum pH of 5.5. This is the first report of a functional characterization of a Type III methyltransferase from *H. pylori*. Interestingly, HP0593 MTase exists as both monomer and dimer in solution but functions as dimer during methylation reaction. HP0593 MTase activity is shown to be stimulated in the presence of divalent metal ions such as Co^2+^, Mg^2+^ and Mn^2+^ and SPR analysis revealed that in presence of Mn^2+^, HP0593 MTase binds to duplex DNA with a higher affinity. The stimulation of MTase activity by Co^2+^ and Mn^2+^ has been reported here for first time in case of a Type III MTases. Preincubation and isotope partitioning analyses revealed that HP0593 MTase-DNA complex is catalytically competent. Initial velocity studies at different AdoMet concentration showed cooperative (sigmoidal) behavior and Hill plot analysis showed that two molecules of AdoMet binds to HP0593 MTase. Two molecules of AdoMet binding may have an implication in increasing the local pool of AdoMet and hence, faster binding and release of the same may enhance the methylation rate. HP0593 MTase methylates DNA containing more than one recognition site in a distributive manner. As HP0593 MTase recognizes and methylates adenine in 5’-GCAG-3’, the probability of occurrence of such a sequence is greater in the genome. The distribution of GCAG sites in 26695 genome has been determined by regulatory sequence analysis tools (RSAT-http://rsat.ulb.ac.be/rsat/). These sites are found at high frequency in the potential promoter region of genes like *flgH*, *ureE*, *cagA*, *hp0017* (*virB4* homologue) and also in the intergenic regions of flagellar and cag pathogenicity island genes. These genes are of great importance in context of physiology of *H. pylori* because they are involved in colonization and pathogenesis processes. Hence, these may represent candidate genes that are regulated by GCAG methylation under acidic condition, where other DNA MTases may not be functional.

## Supporting Information

Figure S1 **Cloning, over-expression and purification of HP0593 protein. A**. PCR amplification of 1797 bp *hp0593* gene from *H. pylori* 26695 genomic DNA, Lane 1, 1 kb DNA ladder; lane 2, control, without genomic DNA; lane 3, *hp0593* gene amplification. **B**. Restriction enzyme digestion of pET14b-*hp0593* clone. Lane 1, DNA alone; lane 2, DNA + NdeI; lane 3, DNA + NdeI and BamHI; lane 4, 1.0 kb DNA ladder, OC = open circular, L = linear, CCC = covalently closed circular. **C**. Over-expression of (His)_6_-HP0593 recombinant protein in *E. coli* BL21 (DE3) pLysS cells. Lane 1, protein molecular weight marker (Fermentas Life- Sciences); lane 2, induced *E. coli* BL21 (DE3) pLysS cells; lane 3, uninduced pET14b vector in *E. coli* BL21 (DE3) pLysS cells; lane 4, induced pET14b vector in *E. coli* BL21 (DE3) pLysS cells, lane 5, uninduced pET14b-*hp0593* plasmid in *E. coli* BL21 (DE3) pLysS cells; lane 6, induced pET14b-*hp0593* plasmid in *E. coli* BL21 (DE3) pLysS cells with 1.0 mM IPTG. **D**. Silver stained 0.1% SDS-10% PAGE gel. Lane 1, purified (His)_6_-HP0593 recombinant protein; lane 2, protein mol. wt. marker (Fermentas Life- Sciences). **E**. MALDI-MS spectrum of purified (His)_6_-HP0593 recombinant protein. **F**. Western blot analysis. Lane 1, BSA (negative control); lane 2, purified (His)_6_-HP0593 recombinant protein; lane 3, pre-stained protein mol. wt. marker (Fermentas Life- Sciences).
(TIF)

Figure S2 **Molecular mass determination of HP0593 protein. A**. Gel filtration chromatography under nondenaturing conditions. Standard curve *Ve/Vo* versus log molecular weight, where *Ve* corresponding to the peak elution volume of the protein and *Vo* representing the void volume of the column determined using Blue dextran (2,000 kDa). 1. Horse myoglobin (17 kDa), 2. Chicken ovalbumin (44 kDa), 3. BSA (66 kDa), 4. EcoP15I MTase (150 kDa), 5. γ-globulin (158 kDa) and 6. Thyroglobulin (670 kDa). (*Inset*) Elution profile of HP0593 (800 µg/ml). **B.** Chemical crosslinking of HP0593 MTase with glutaraldehyde: HP0593 MTase (2.0 µM) was incubated with 0.01%–0.08% of glutaraldehyde (final concentration) at 4°C for 10 min. Reactions were stopped by adding SDS-loading buffer and boiled for three min at 100°C. The reaction mixtures were analyzed on a 10% polyacrylamide gel containing 0.1% SDS. The gel was stained with silver nitrate. Lane 1, HP0593 MTase alone; lanes 2–5, HP0593 MTase and 0.01%–0.08% of glutaraldehyde; lane 6, EcoP15I MTase alone; lane 7, EcoP15I MTase + 0.06% glutaraldehyde; lane 8, protein molecular weight marker. D, dimer; M, monomer.
(TIF)

Figure S3 **Target base for methylation.** **A.** Native polyacrylamide gel (6%) showing cleavage pattern of duplex 7 DNA. Lane 1, duplex 7 DNA; lane 2, duplex 7 DNA + R.HhaI; lane 3, HP0593 MTase methylated duplex 7 DNA + R.HhaI; and lane 4, HhaI MTase methylated duplex 7 DNA + R.HhaI. **B.** Native polyacrylamide gel (6%) showing cleavage pattern of duplex 8 DNA. Lane 1, Low molecular weight DNA ladder; lane 2, duplex 8 DNA; lane 3, duplex 8 DNA + R.AluI; lane 4, HP0593 MTase methylated duplex 8 DNA + R.AluI. **C.** Native polyacrylamide gel (6%) showing cleavage pattern of duplex 9 DNA. Lane 1, Low molecular weight DNA ladder; lane 2, duplex 9 DNA; lane 3, duplex 9 DNA + R.PstI; lane 4, HP0593 MTase methylated duplex 9 DNA + R.PstI. **D.** Methylation activity of HP0593 MTase with unmethylated (duplex 10) and methylated duplex DNAs (duplexes 11–15).
(TIF)

Figure S4 **Mode of methylation of HP0593 MTase on DNA containing two recognition sites.** HP0593 MTase (150 nM) was incubated with 2 µM biotin conjugated duplex 16 at 25°C for 5 min to facilitate formation of the HP0593–DNA catalytic complex. The reaction mixture was divided into two sets, to one set- reaction was started by adding 1.5 µM of [^3^H] AdoMet (•) and incorporation of methyl group was monitored by biotin-avidin microplate assay. To the other set reaction was started by adding 20 µM unlabeled duplex 17 as trap along with 1.5 µM of [^3^H] AdoMet as described in the materials and methods. Aliquots were withdrawn at the indicated time points. The incorporation of methyl groups was measured by either Biotin-avidin microplate assay (**▪**), or in the control reaction methylation was measured by the DE81 filter binding assay (**▴**). The experiment was carried out in duplicates and data was plotted using GraphPad Prism 5. [Bt]  =  biotinylation.
(TIF)

Figure S5 **DNA binding by HP0593 MTase.** **A**. Binding of HP0593 MTase to duplex 2 DNA. Lane 1, radiolabeled duplex 2; lanes 2–9, increasing concentrations of HP0593 MTase (0.5 µ µM-5.0 µM), were incubated with 5′ [γ*-^32^P*] end-labeled duplex 2 (approximately 100 nM) in methylation buffer on ice for 10 min and analyzed as described in materials and methods; lanes 10–13, chase with excess of unlabeled duplex 2 DNA (5, 10,15, and 20-fold, respectively). **B.** Binding in presence of 10 µM sinefungin. Lane 1, radiolabeled duplex 2; lanes 2–9, increasing HP0593 MTase (0.5 µM–5.0 µM); lanes 10–13, chase with excess of unlabeled duplex 2 DNA (5, 10, 15, and 20-fold, respectively). **C**. Binding of HP0593 MTase to non-specific duplex 19 DNA: Lane 1, radiolabeled duplex 19; lanes 2–3, radiolabeled duplex 19 + HP0593 (0.5 and 3.0 µM); lanes 4–5, radiolabeled duplex 19 + HP0593 (0.5 and 3.0 µM respectively) + 5 µM AdoHcy; lanes 6–7, radiolabeled duplex 19 + HP0593 (0.5 and 3.0 µM respectively) + 10 µM sinefungin. CI  =  complex 1, CII  =  complex 2.
(TIF)
