# Supplementary figures and images for: Functional Analysis of an Acid Adaptive DNA Adenine Methyltransferase from Helicobacter pylori 26695
Source: PLoS One. 2011 Feb 9;6(2):e16810. doi: 10.1371/journal.pone.0016810 (PMC3036652; doi:10.1371/journal.pone.0016810)

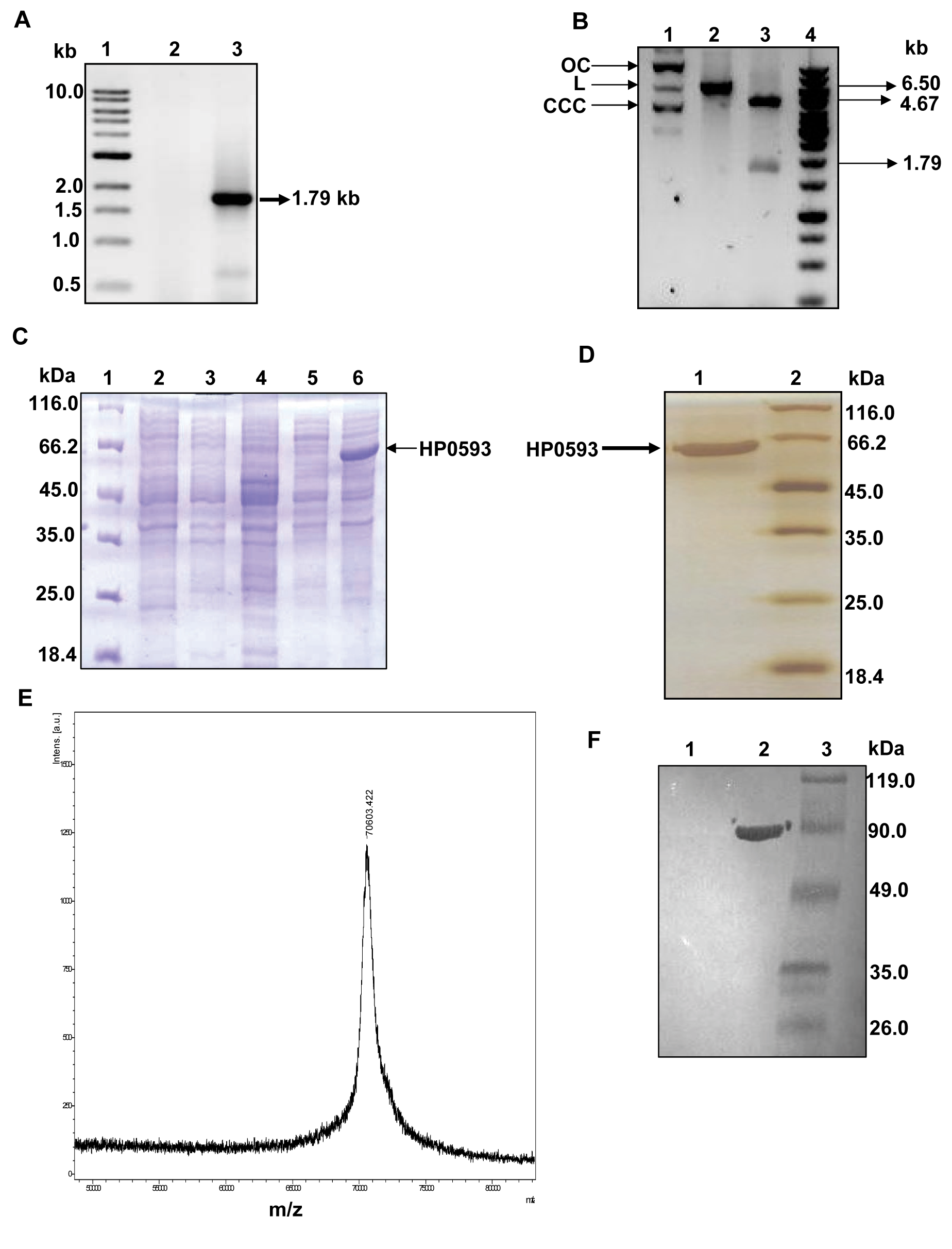

Supplement: Figure S1 — Cloning, over-expression and purification of HP0593 protein. A. PCR amplification of 1797 bp hp0593 gene from H. pylori 26695 genomic DNA, Lane 1, 1 kb DNA ladder; lane 2, control, without genomic DNA; lane 3, hp0593 gene amplification. B. Restriction enzyme digestion of pET14b-hp0593 clone. Lane 1, DNA alone; lane 2, DNA + NdeI; lane 3, DNA + NdeI and BamHI; lane 4, 1.0 kb DNA ladder, OC = open circular, L = linear, CCC = covalently closed circular. C. Over-expression of (His)6-HP0593 recombinant protein in E. coli BL21 (DE3) pLysS cells. Lane 1, protein molecular weight marker (Fermentas Life- Sciences); lane 2, induced E. coli BL21 (DE3) pLysS cells; lane 3, uninduced pET14b vector in E. coli BL21 (DE3) pLysS cells; lane 4, induced pET14b vector in E. coli BL21 (DE3) pLysS cells, lane 5, uninduced pET14b-hp0593 plasmid in E. coli BL21 (DE3) pLysS cells; lane 6, induced pET14b-hp0593 plasmid in E. coli BL21 (DE3) pLysS cells with 1.0 mM IPTG. D. Silver stained 0.1% SDS-10% PAGE gel. Lane 1, purified (His)6-HP0593 recombinant protein; lane 2, protein mol. wt. marker (Fermentas Life- Sciences). E. MALDI-MS spectrum of purified (His)6-HP0593 recombinant protein. F. Western blot analysis. Lane 1, BSA (negative control); lane 2, purified (His)6-HP0593 recombinant protein; lane 3, pre-stained protein mol. wt. marker (Fermentas Life- Sciences). (TIF) [file pone.0016810.s001.tif]

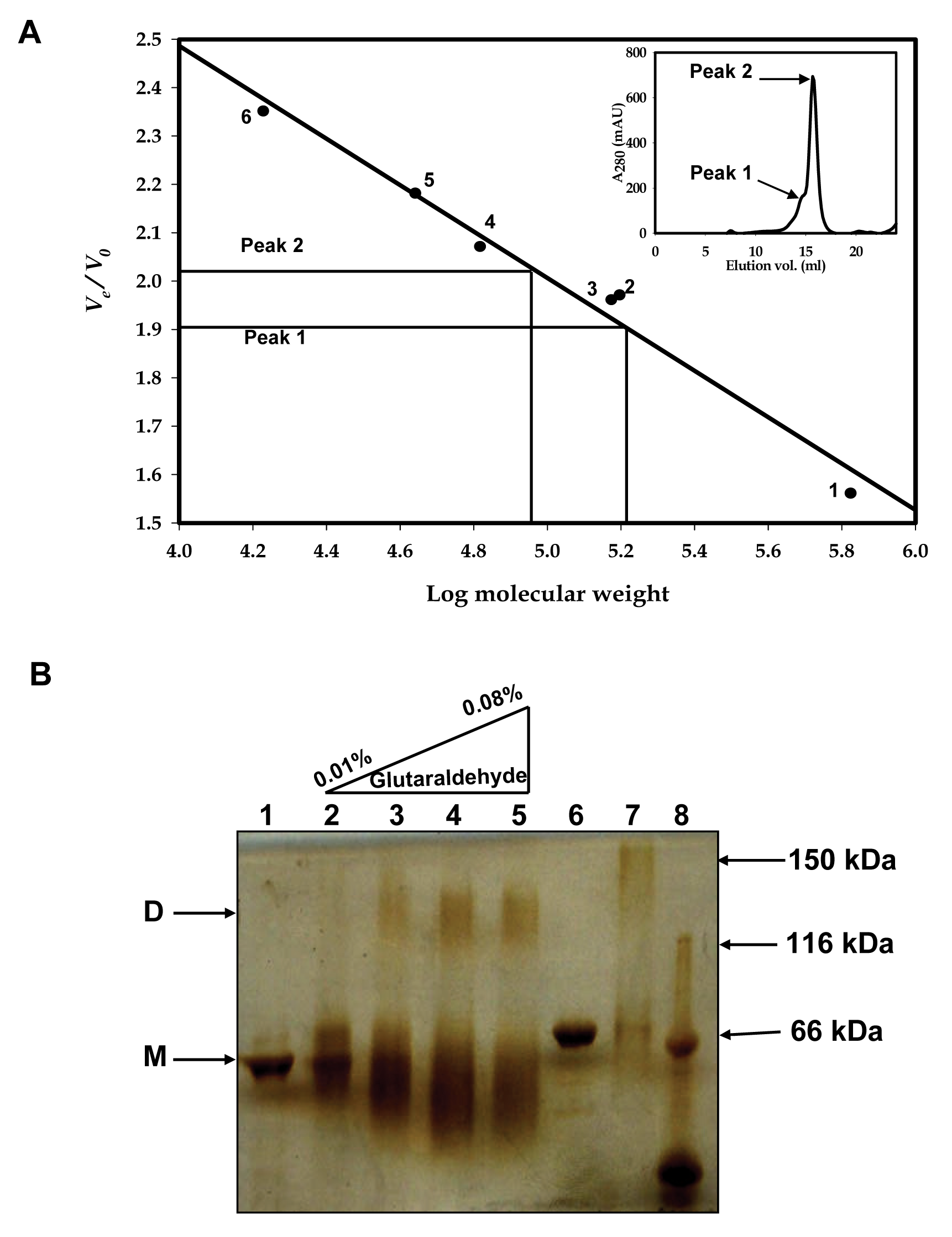

Supplement: Figure S2 — Molecular mass determination of HP0593 protein. A. Gel filtration chromatography under nondenaturing conditions. Standard curve Ve/Vo versus log molecular weight, where Ve corresponding to the peak elution volume of the protein and Vo representing the void volume of the column determined using Blue dextran (2,000 kDa). 1. Horse myoglobin (17 kDa), 2. Chicken ovalbumin (44 kDa), 3. BSA (66 kDa), 4. EcoP15I MTase (150 kDa), 5. γ-globulin (158 kDa) and 6. Thyroglobulin (670 kDa). (Inset) Elution profile of HP0593 (800 µg/ml). B. Chemical crosslinking of HP0593 MTase with glutaraldehyde: HP0593 MTase (2.0 µM) was incubated with 0.01%–0.08% of glutaraldehyde (final concentration) at 4°C for 10 min. Reactions were stopped by adding SDS-loading buffer and boiled for three min at 100°C. The reaction mixtures were analyzed on a 10% polyacrylamide gel containing 0.1% SDS. The gel was stained with silver nitrate. Lane 1, HP0593 MTase alone; lanes 2–5, HP0593 MTase and 0.01%–0.08% of glutaraldehyde; lane 6, EcoP15I MTase alone; lane 7, EcoP15I MTase + 0.06% glutaraldehyde; lane 8, protein molecular weight marker. D, dimer; M, monomer. (TIF) [file pone.0016810.s002.tif]

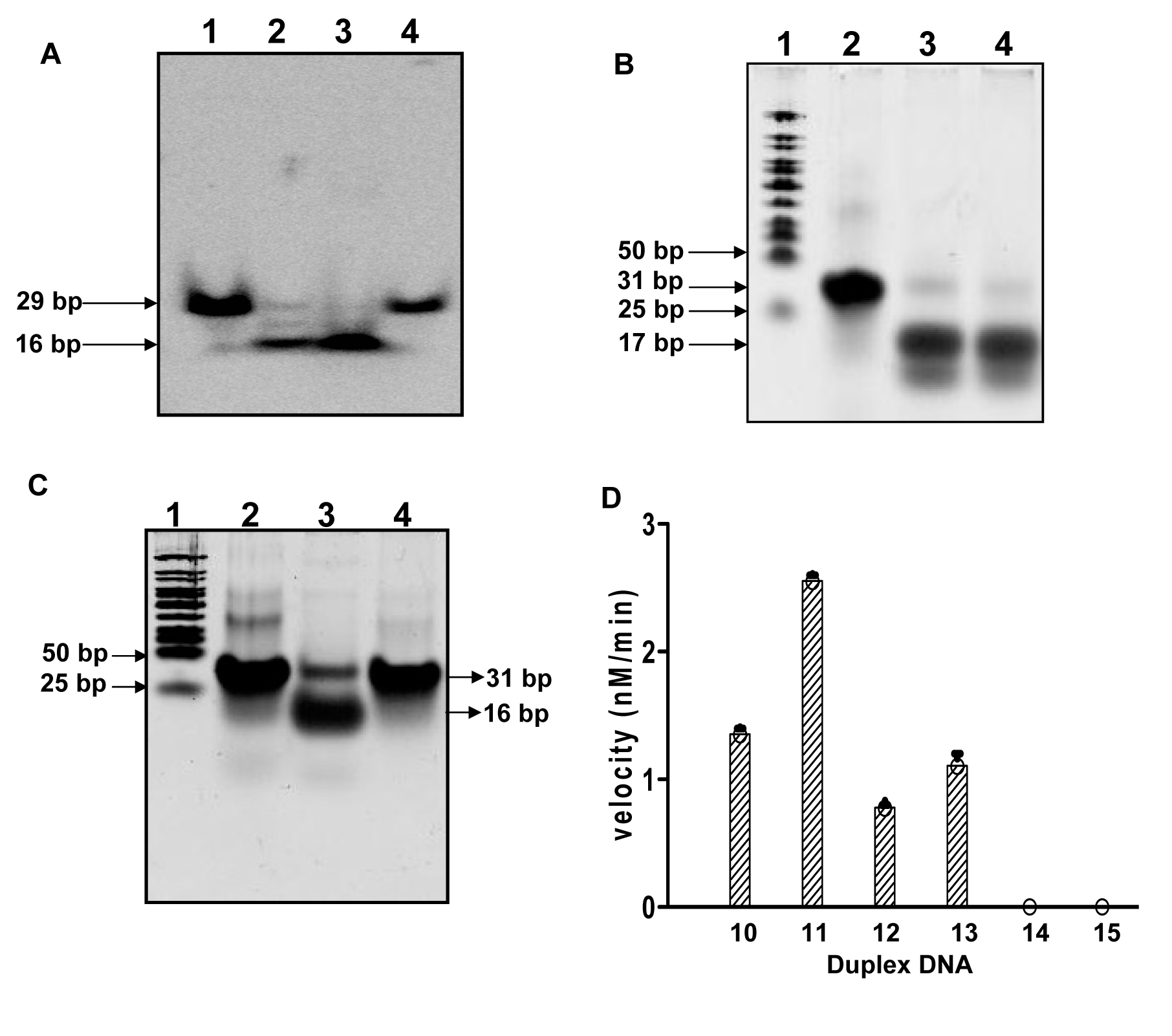

Supplement: Figure S3 — Target base for methylation. A. Native polyacrylamide gel (6%) showing cleavage pattern of duplex 7 DNA. Lane 1, duplex 7 DNA; lane 2, duplex 7 DNA + R.HhaI; lane 3, HP0593 MTase methylated duplex 7 DNA + R.HhaI; and lane 4, HhaI MTase methylated duplex 7 DNA + R.HhaI. B. Native polyacrylamide gel (6%) showing cleavage pattern of duplex 8 DNA. Lane 1, Low molecular weight DNA ladder; lane 2, duplex 8 DNA; lane 3, duplex 8 DNA + R.AluI; lane 4, HP0593 MTase methylated duplex 8 DNA + R.AluI. C. Native polyacrylamide gel (6%) showing cleavage pattern of duplex 9 DNA. Lane 1, Low molecular weight DNA ladder; lane 2, duplex 9 DNA; lane 3, duplex 9 DNA + R.PstI; lane 4, HP0593 MTase methylated duplex 9 DNA + R.PstI. D. Methylation activity of HP0593 MTase with unmethylated (duplex 10) and methylated duplex DNAs (duplexes 11–15). (TIF) [file pone.0016810.s003.tif]

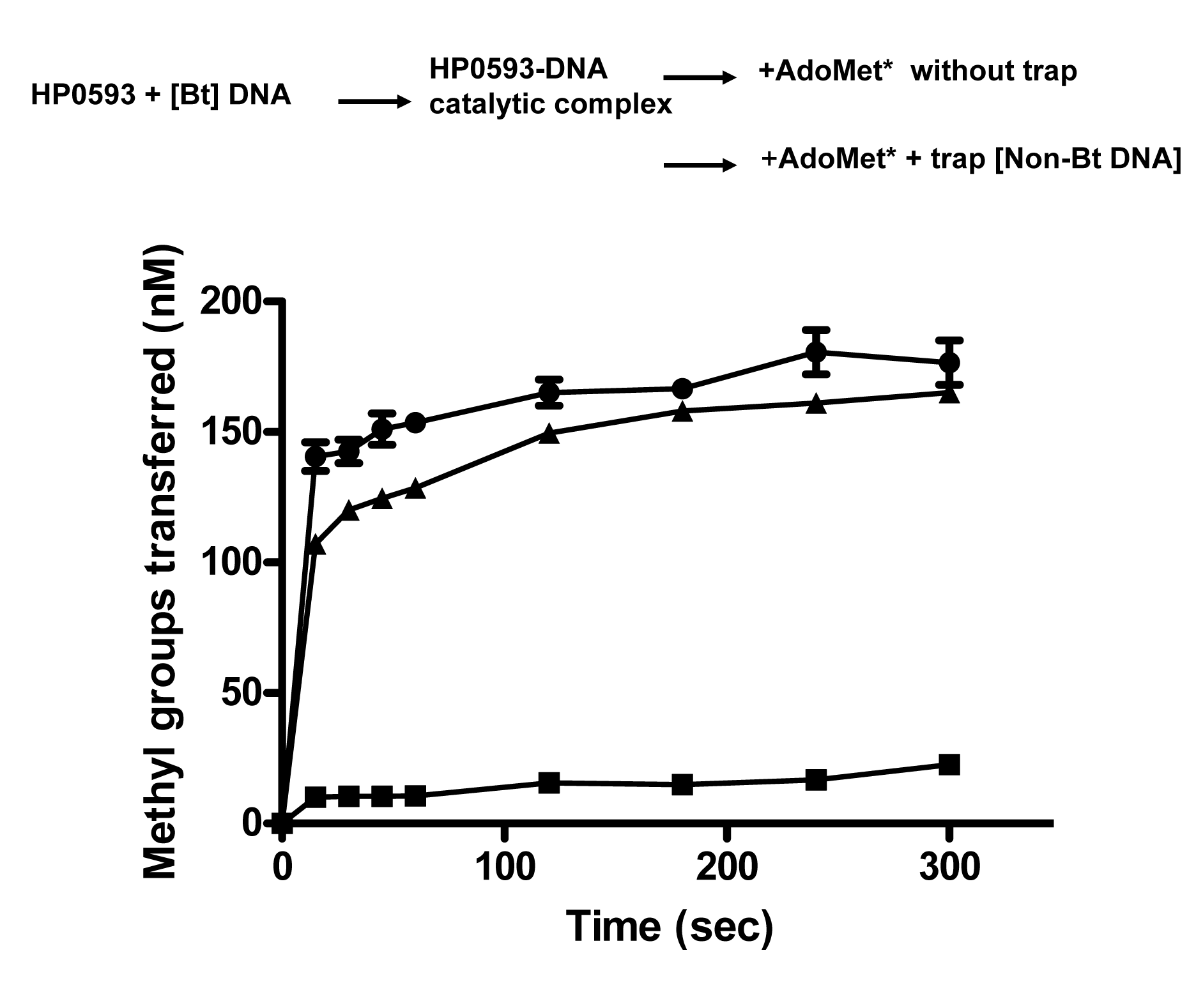

Supplement: Figure S4 — Mode of methylation of HP0593 MTase on DNA containing two recognition sites. HP0593 MTase (150 nM) was incubated with 2 µM biotin conjugated duplex 16 at 25°C for 5 min to facilitate formation of the HP0593–DNA catalytic complex. The reaction mixture was divided into two sets, to one set- reaction was started by adding 1.5 µM of [3H] AdoMet (•) and incorporation of methyl group was monitored by biotin-avidin microplate assay. To the other set reaction was started by adding 20 µM unlabeled duplex 17 as trap along with 1.5 µM of [3H] AdoMet as described in the materials and methods. Aliquots were withdrawn at the indicated time points. The incorporation of methyl groups was measured by either Biotin-avidin microplate assay (▪), or in the control reaction methylation was measured by the DE81 filter binding assay (▴). The experiment was carried out in duplicates and data was plotted using GraphPad Prism 5. [Bt] = biotinylation. (TIF) [file pone.0016810.s004.tif]

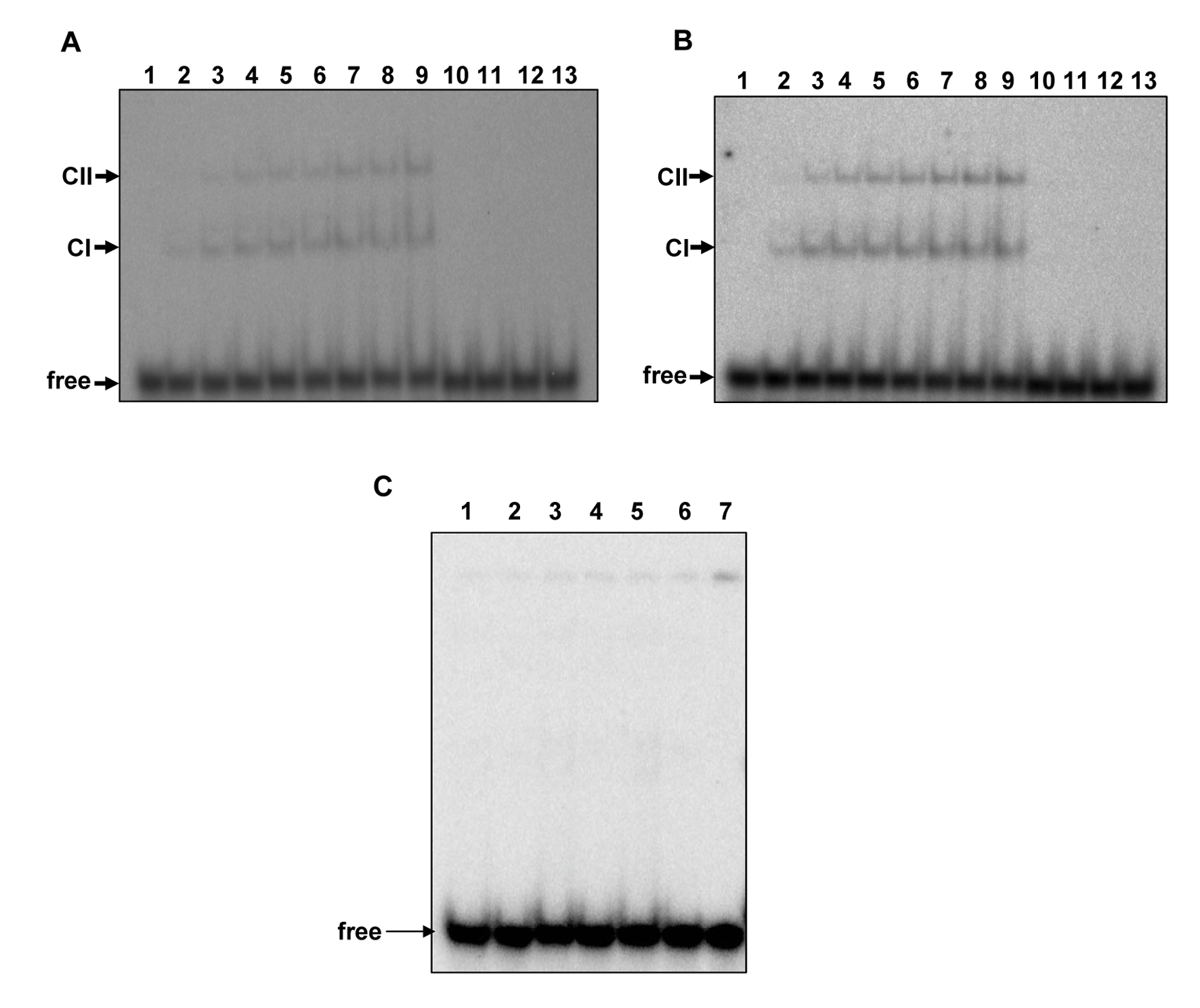

Supplement: Figure S5 — DNA binding by HP0593 MTase. A. Binding of HP0593 MTase to duplex 2 DNA. Lane 1, radiolabeled duplex 2; lanes 2–9, increasing concentrations of HP0593 MTase (0.5 µ µM-5.0 µM), were incubated with 5′ [γ-32P] end-labeled duplex 2 (approximately 100 nM) in methylation buffer on ice for 10 min and analyzed as described in materials and methods; lanes 10–13, chase with excess of unlabeled duplex 2 DNA (5, 10,15, and 20-fold, respectively). B. Binding in presence of 10 µM sinefungin. Lane 1, radiolabeled duplex 2; lanes 2–9, increasing HP0593 MTase (0.5 µM–5.0 µM); lanes 10–13, chase with excess of unlabeled duplex 2 DNA (5, 10, 15, and 20-fold, respectively). C. Binding of HP0593 MTase to non-specific duplex 19 DNA: Lane 1, radiolabeled duplex 19; lanes 2–3, radiolabeled duplex 19 + HP0593 (0.5 and 3.0 µM); lanes 4–5, radiolabeled duplex 19 + HP0593 (0.5 and 3.0 µM respectively) + 5 µM AdoHcy; lanes 6–7, radiolabeled duplex 19 + HP0593 (0.5 and 3.0 µM respectively) + 10 µM sinefungin. CI = complex 1, CII = complex 2. (TIF) [file pone.0016810.s005.tif]
